# From methodological limitations to the function of metallothioneins - a guide to approaches for determining weak, moderate, and tight affinity zinc sites

**DOI:** 10.1093/mtomcs/mfad027

**Published:** 2023-04-27

**Authors:** Adam Pomorski, Agnieszka Drozd, Anna Kocyła, Artur Krężel

**Affiliations:** Department of Chemical Biology, Faculty of Biotechnology, University of Wrocław, Joliot-Curie 14a, 50-383 Wrocław, Poland; Department of Chemical Biology, Faculty of Biotechnology, University of Wrocław, Joliot-Curie 14a, 50-383 Wrocław, Poland; Department of Chemical Biology, Faculty of Biotechnology, University of Wrocław, Joliot-Curie 14a, 50-383 Wrocław, Poland; Department of Chemical Biology, Faculty of Biotechnology, University of Wrocław, Joliot-Curie 14a, 50-383 Wrocław, Poland

**Keywords:** dissociation constant, zinc probe, mass spectrometry, competition, metal binding

## Abstract

Mammalian metallothioneins (MTs) are small cysteine-rich proteins whose primary role is participation in zinc and copper homeostasis. Ever since their discovery, MTs have been investigated in terms of metal-binding affinity. The initial concept of seven Zn(II) ions (Zn_7_MT) bound with the same, undifferentiated low-picomolar affinity in the α and β domains prevailed for many years and derived from spectroscopic studies. The application of fluorescent zinc probes has changed the perception of MTs, showing that they function in nanomolar to subnanomolar free zinc concentrations due to the presence of tight, moderate, and weak binding sites. The discovery of Zn(II)-depleted MTs in many tissues and determination of cellular free Zn(II) concentrations with differentiated zinc affinity sites revealed the critical importance of partially saturated Zn_4–6_MTs species in cellular zinc buffering in a wide picomolar to nanomolar range of free Zn(II) concentrations. Until today, there was no clear agreement on the presence of differentiated or only tight zinc sites. Here, we present a series of spectroscopic, mass spectrometry-based, and enzymatic competition experiments that reveal how weak, moderate, or high-affinity ligands interact with human MT2, with special attention to the determination of Zn(II) affinities. The results show that the simplification of the stability model is the major reason for determining significantly different stability data that obscured the actual MTs function. Therefore, we emphasize that different metal affinities are the single most important reason for their presumed function, which changed over the years from tight binding and, thus, storage to one that is highly dynamic.

## Graphical Abstract

**Graphical Abstract:**
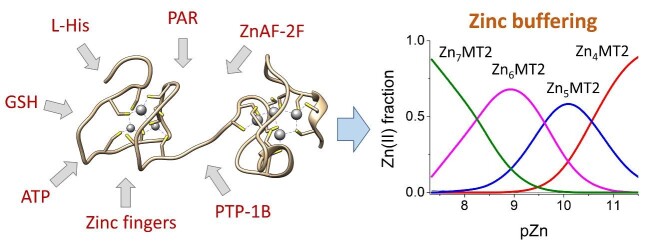
We demonstrate that the simplification of the stability model is the major reason for determining significantly different stability data that obscured the understanding of MTs functions. We emphasize that different metal affinities are the most important reason for their presumed function, which changed over the years from tight binding and, thus, storage to one that is highly dynamic.

## Introduction

**Fig. 1:**
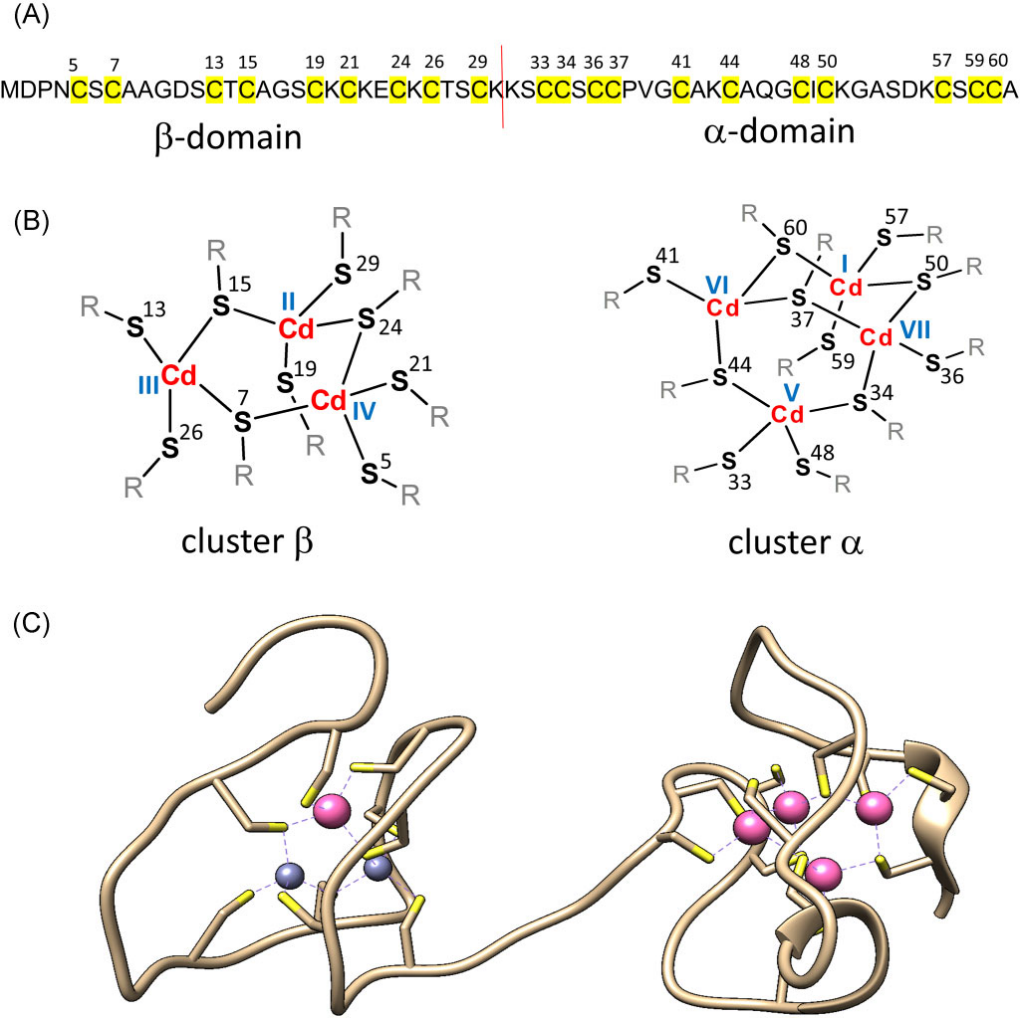
Sequence and structure of mammalian MT2. (A) Sequence of human MT2 used in this study. Yellow color and red line highlight cysteinyl residues responsible for metal binding and border between α and βdomains, respectively. (B) Structures of Cd_3_S_9_ (β) and Cd_4_S_11_ (α) clusters in rabbit MT2 (rabMT2). Metal-binding sites were assigned to coordinating residues based on NMR studies.^18,23^ (C) The only crystal structure of hepatic rat MT2 as a mixed complex Cd_5_Zn_2_MT2.^16^ (PDB ID: 4MT2)

Mammalian metallothioneins (MTs) are a family of small Cys-rich proteins present in four major isoforms (MT1–MT4) that participate in Zn(II) and Cu(I) homeostasis.^1,2^ MTs also bind toxic metal ions such as Cd(II), Pb(II), Hg(II), and others, limiting their negative effects on cells.^2–5^ They participate in numerous redox reactions protecting cells against oxidative stress and take part in neuroprotective mechanisms.^2,6–8^ MTs are present mostly in cytosol, but they have also been found in the nucleus, mitochondria, and extracellular environment.^2,7,9,10^ MT1 (and its subisoforms) and MT2 lack tissue specificity and are expressed in all kinds of cells at different levels and ratios, whereas MT3 and MT4 have been found mostly in the central nervous system and in stratified epithelial cells, respectively.^2,7,11–15^ Mammalian MTs contain two distinct regions: the C-terminal α-domain with the M_4_(Cys)_11_ cluster and the N-terminal β-domain with the M_3_(Cys)_9_ cluster (Fig. 1A and B)—although the term ‘domain’ does not fully apply here. Until today, the only crystal structure solved was the hepatic rat Cd_5_Zn_2_MT2 (Fig. 1C).^16^ However, the structures of isolated human, rabbit and rat Cd(II)-containing α- and β-MT2 domains have been determined in NMR studies and continued later for MT1 and MT3.^17–22^ The lack of NMR structures of Zn(II)-loaded domains [or full Zn(II)-loaded protein] was caused by the poor nuclear properties of zinc isotopes that do not allow Zn(II)-cysteinyl residue assignment, as in the case of ^111^Cd and ^113^Cd.^23^ Therefore, current structural knowledge on Zn(II)-MTs still relies on mixed Zn(II)/Cd(II) rat MT2 or Cd(II)-loaded domains. The last two decades have brought progress in the application of mass spectrometry (MS) approaches for structural characterization of MTs. With the support of molecular dynamics (MD), they afforded deeper insights into Zn(II) and Cd(II) [also Ag(I), Cu(I), and other cations] folding/unfolding processes and structure of partially metal-loaded species, surpassing all previous methods.^2,4,24–30^ Importantly, these MS and MD-based approaches made it possible to map Zn(II)-binding sites of the highest, medium, and weak affinities (only order, without numerical values), which is not without importance for the biological role of these proteins in cellular zinc handling and buffering processes.^30–33^ According to that, four tight Zn(II) ions of MT2 are located in both domains, binding to a high number of Cys residues. Remaining three Zn(II) ions with moderate and weak affinity complete both clusters.^30,33^ However, the weakest zinc site was identified in the β-domain, indicating it is different from the α-domain character.^27–30^ Differentiated affinities of Zn(II) ions in MTs in the light of cellular free Zn(II) concentration range and findings of partially saturated MT fractions in tissues show that Zn_4–6_MT species (partially depleted or partially saturated), not Zn_7_MT, play a critical role in zinc buffering, which operates in relatively wide picomolar to nanomolar range.^1,2,31,32^ It must be underlined that each partially metallated Zn(II)-MTs species has its own structure, which differs from that of fully metallated and well-known Zn_7_MT.^30,33^ The heterogeneous structural and thermodynamic character of MTs is fundamental for supporting cellular metalloproteins of various structures and affinity with their metal ions or accepting zinc surplus.^2^

Investigation of MTs and overall knowledge regarding their biophysical and biochemical properties require the determination of essential and toxic metal-to-protein affinities, which are critical for understanding metal binding/dissociation equilibria and overall mechanisms of action. Due to the lack of MTs’ secondary structure, multiple metal ions’ binding, and high content of cysteinyl residues, the determination of stability constants is no trivial task. Early studies on Zn(II) and Cd(II) interaction with MTs demonstrated that the metalation process is pH-reversible and can be investigated by absorption spectroscopy in the UV range due to (dis)appearance of characteristic ligand-to-metal charge transfer (LMCT) bands. Assuming their intensity change was proportional to metal load, average binding, or dissociation constants (*K*_b _= 1/*K*_d_) were determined.^34,35^ This assumption sets the trend for future analysis of MTs. For Zn(II), the observable isotherm indicated one transition event, and assuming the same p*K*_a_ value of all cysteinyl thiols of 8.9, an average *K*_d_ (*K*_d_^av^) of 10^−13^–10^−12^ M was found (Table 1).^36–41^ In the case of Cd(II) (un)binding, two events are detectable for all investigated mammalian MTs, and *K*_d_^av^ values are ∼10^−13^ and ∼10^−15^ M for the first and second event, respectively.^13,34,42^ These two events for Cd(II) were assumed to sequential domain loading, first to α-domain and then to β-domain.^13,34,42,43^ This scenario has been validated, but our recent studies have indicated structural differences in mechanisms of Cd(II) and Zn(II) metalation of MT2.^30^ The observation of a single event in the pH-dependent isotherm for Zn(II) stems from the low sensitivity of the applied spectroscopic studies, which does not allow differences in Zn(II) stability constants to be distinguished. For example, a Zn(II)-sensitive chelating probe such as thiosemicarbazone KTSM_2_ showed that Zn(II) ions bind to MT1 and MT2 with *K*_d_^av^ varying from 10^−12^ to 10^−11^ M, depending on the isoform, without differences between particular metal ions (Table 1).^38,40^ The competition with chromophoric PAR or non-chromophoric chelating agents (e.g. nitrilotriacetic acid NTA) was convergent with previous studies indicating all Zn(II) ions binding with the same (or undistinguished) affinities (Table 1).^38,40,44,45^ However, several other experiments, mostly based on enzymatic assays, have shown that Zn(II) can be efficiently transferred from a fully loaded Zn_7_MT molecule to proteins with Zn(II)-binding sites of moderate affinity. The incubation of Zn_7_MT with the apoforms of sorbitol dehydrogenase (SDH) (–log*K*_d_^Zn^ = 11.2) and carboxypeptidase A (–log*K*_d_^Zn^ = 8.3) caused rapid recovery of enzyme activity due to the fast transfer of one Zn(II) ion from metallothionein.^46–48^ This is in opposition to the statement that MTs bind all Zn(II) ions with the same low or subpicomolar affinity. It was confirmed later by competition with ultra-sensitive zinc fluorescent probes (FluoZin-3 and Rhod-Zin-3), which indicated that seven Zn(II) ions of human MT2 are bound with *K*_d_ varying from ∼10^−12^ to ∼10^−8^ M (picomolar to nanomolar affinity).^32^ A similar conclusion came from isothermal titration calorimetry (ITC) investigation of human Zn(II)-MT3.^49^ These affinities validate the cellular buffering character of MTs and their central role in cellular Zn(II) distribution. However, it is neglected by some investigators. For instance, competition of metal-free human MT1a and metal-free carbonic anhydrase (CA) for Zn(II) studied in ESI-MS titration showed that seven Zn(II) ions bind to MT1a sequentially with –log*K*_d1–7_ in the range from 11.3 to 13.^50^ These data indicate that affinity for Zn(II) is indeed differentiated but occurs in a significantly different and narrowed range, not corresponding to cellular zinc fluctuations, which changes perception of MTs function. However, we recently observed that the elevation of binding constants determined by ESI-MS in comparison to water is related to differently (de)hydrated Zn(II)-loaded MT species (Zn_1–7_MT2) in the gas phase and buffered water medium, which demonstrate different intramolecular stabilization effects pattern.^30,33^

**Table 1.** Comparison of apparent average dissociation constants (*K*_d_^av^) of Zn(II) mammalian MTs determined over the decades using various methods and conditions. r, m, and h before a particular MT isoform refer to rabbit, mouse, and human protein, respectively

Method of determination	Conditions of determination	Dissociation constant *K*_d_^av^ or *K*_d1–7_ (M)	Reference
Spectroscopic (UV range-monitored pH-titration)	pH 7.0	5 × 10^−13^	[37]
Spectroscopic competition with H_2_KTSM, NTA	25°C, 0.1 M KCl, pH 7.4	5.9 × 10^−12^ (rMT1, rMT2)	[38, 40]
Differential pulse polarography (DPP)	25 mM Tris–HCl, pH 7.5	1.3 × 10^−12^ (rMT1) 7.9 × 10^−13^ (rMT2)	[39]
^19^F-NMR—competition with 5F-BAPTA	10 mM Tris–HCl pH 8.0	3.2 × 10^−12^ (rMT2) 1.6 × 10^−11^ (hMT3)	[41]
Fluorimetry—competition with FluoZin-3/RodZin-3	50 mM Na^+^-HEPES, 0.1 M KNO_3_, pH 7.4	(hMT2) 1.6 × 10^−12^ (4 sites) 3.6 × 10^−11^ (1 site) 1.1 × 10^−10^ (1 site) 2 × 10^−8^ (1 site)	[32]
PAR competition	25°C, 25 mM Tris–HCl, 50 mM NaCl, pH 7.4	3.1 × 10^−11^ (MT2) 1.3 × 10^−10^ (MT3)	[45]
ITC—competition with EDTA	25°C, 100 mM MES pH 6.0	(mMT3) 1.5 × 10^−11^ (4 sites) ∼4 × 10^−10^ (2 sites) ∼1 × 10^−8^ (1 site)	[49]
ITC—competition with DTPA	25°C, pH 7.4 (buffer independent)	2.5 × 10^−12^ (mMT3)	[51]
ESI-MS—competition with CA	Gas phase	(hMT1) 4.5 × 10^−13^, 3.4 × 10^−13^, 3.0 × 10^−13^, 4.3 × 10^−13^, 6.2 × 10^−13^, 8.9 × 10^−13^, 1.6 × 10^−12^	[50]
ESI-MS	Gas phase	(hMT2) 1.3 × 10^−12^, 1.0 × 10^−12^, 1.6 × 10^−12^, 2.0 × 10^−12^, 2.0 × 10^−11^, 1.3 × 10^−11^, 2.5 × 10^−11^	[32]

Due to the ongoing discussion on the stability of Zn(II)-MTs systems, particularly the presence or absence of weak and moderate bound zinc sites and related biological consequences, the main goal of this study is to provide additional evidence of Zn(II) to MT affinities and discuss approaches to their determination from various perspectives. Therefore, here, we utilized several approaches with UV-vis or fluorescent probes of various Zn(II) affinities and confidence ranges. We monitored either direct metal-to-protein interaction or competition between metal-free (thionein, T) or fully loaded Zn_7_MT2. We also show and discuss how Zn(II) transfer occurs between Zn(II)-binding proteins and the dynamic Zn_4–7_MT system. We put special attention on stoichiometric or model assumptions that are frequently made during stability constant determination and show how they may affect the results in final data interpretation. We also underline that the MT production method, bacterial recombination or isolation from tissues, may be a reason for different results. Finally, we present a workflow scheme that should be undertaken in the characterization of new (not previously studied) metallothioneins from various organisms or other Cys-rich or MT-like proteins, which bind several metal ions but do not demonstrate highly ordered structures. Overall, we show how the limitations of applied methodology affect our perception of MTs function, which has changed from tight metal binding and, thus, storage to one that is highly dynamic.^32^

## Experimental procedures

### Synthesis of zinc finger peptides

Zinc fingers (ZFs): ZScan20-2, ZNF442, consensus ZF CP1 (CCHH), ZF133-11, and its C7E mutant (ZF133-11 C7E) with weaker Zn(II) affinity were synthesized on TentaGel R RAM resin (loading 0.19 mmol/g, Rapp Polymere GmbH) using a Liberty 1 microwave-assisted synthesizer (CEM, USA) according to the standard Fmoc strategy described in detail before.^52,53^ The full sequences are given in Table S1. All peptides were acetylated at the N-terminus and purified by HPLC (Dionex Ultimate 3000 system) on a Phenomenex C18 column (Gemini-NX 5 µm, 110 Å) using a gradient of 95% acetonitrile/5% water (0.1% TFA) with 0.1% TFA in water, as described previously.^53^ After purification, peptides were lyophilized. The identity of the obtained pure peptides was confirmed by ESI-MS (API 2000, Applied Biosystems, USA). The list of theoretical and experimental masses is presented in Table S2.

### Expression and purification of human MT2

A bacterial expression vector (#105693 Addgene) encoding human MT2 in fusion with chitin-binding protein and the intein-cleavage site was used to transform chemicompetent *E. coli* BL21 DE3 RIL cells.^27^ The culture medium [1.1% (w/v) tryptone, 2.2% (w/v) yeast extract, 0.45% (w/v) glycerol, 1.3% (w/v) K_2_HPO_4_, and 0.38% (w/v) KH_2_PO_4_] was prepared according to previous protocols. Transformed bacteria were cultured at 37°C with shaking until OD_600_ reached 0.5. The induction was performed with 0.1 mM IPTG, and the culture was incubated overnight at 20°C with vigorous shaking. All subsequent steps of purification were conducted at 4°C. Cells were collected by centrifugation at 4000× *g* for 10 min, suspended in 50 ml of cold buffer with 20 mM Na^+^-HEPES, pH 8.0, 500 mM NaCl, 1 mM EDTA (ethylenediaminetetraacetic acid), and 1 mM TCEP (tris(2-carboxyethyl)phosphine hydrochloride), and sonicated for 45 min, followed by centrifugation at 16 000× *g* for 45 min. The supernatant was incubated with 20 ml of chitin resin overnight with mild shaking.^54^ After incubation, the resin was washed 5–6 times with 50 ml of the same buffer. To start the cleavage reaction, 25 ml of buffer containing 20 mM Na^+^-HEPES, pH 8.0, 500 mM NaCl, 1 mM EDTA, and 100 mM DTT (DL-dithiothreitol) was added to the resin, and the mixture was incubated for 48 h at room temperature with mild mixing on the roller.^32^ Cleavage progress was monitored by SDS-PAGE (sodium dodecyl sulfate–polyacrylamide gel electrophoresis). To ensure full reduction of the metallothionein, 50 mg of DTT was added to the solution in the column 30 min before elution. The eluted solution was acidified to pH ∼ 2.5 with 7% HCl and subsequently concentrated to a small volume using Amicon Ultra-4 (3 K MWCO) Centrifugal Filter Units (Merck Millipore, Burlington, MA, USA). Concentrated solution was applied on a SEC-70 gel filtration column (Bio-Rad, Hercules, CA, USA) equilibrated with 10 mM HCl.^55^ The identity of apo*-*MT2 was confirmed by ESI-MS, utilizing an API 2000 instrument (Applied Biosystems) (Table S2). Purified apo-MT2 was not frozen and was used immediately for the experiments or reconstitution with Zn(II) due to its rapid oxidation. In order to visualize proteins during the particular steps of production and purification, we used the biarsenical probe F4FlAsH-EDT_2_, which binds to Cys-rich regions of the protein, emitting green light when excited at 492 nm.^56^

### Reconstitution of human MT2 with Zn(II)

Metallothionein was reconstituted with Zn(II) ions in two different ways. In the first one, it was obtained in the reaction with an 8.5 molar excess of ZnSO_4_ over apo-MT2 under a nitrogen blanket and pH adjusted to 8.6 with small aliquots of 1 M Tris base, as described elsewhere.^57^ Samples were concentrated with Amicon Ultra-4 Centrifugal Filter Units with a membrane cut-off of 3 kDa (Merck Millipore) and purified on a ENrich SEC-70 gel filtration column (Bio-Rad) equilibrated with 20 mM Tris–HCl buffer, pH 8.6 (Bio-Rad NGC).^27^ In the second approach, Zn(II)-loaded MT2 was obtained in such a way as to avoid protein acidification. To do so, apoprotein after DTT-induced cleavage from the resin was centrifuged using Amicon Ultra-4 Centrifugal Filter Units (3 kDa cut-off) to exchange Na^+^-HEPES buffer to 20 mM Tris–HCl, pH 8.6, and 5 mM TCEP using Amicon Ultra-4 3 kDa. Six cycles were performed to ensure the removal of EDTA added to the cleavage buffer. Next, ZnSO_4_ was added at ∼9 molar excess to saturate apoprotein with metal. Finally, MT2 reconstituted at a slightly basic pH was purified by isocratic elution on an ENrich SEC-70 column (Bio-Rad) as reconstituted acidified (first approach) protein with 20 mM Tris–HCl, pH 8.6. Regardless of the method of obtaining holoprotein, the concentration of thiolates and Zn(II) was determined spectroscopically using DTNB (5,5′-dithiobis(2-nitrobenzoic acid)) and PAR (4-(2-pyridylazo)resorcinol) assays, respectively.^58,59^ The concentration of Zn(II) was independently analyzed by inductively coupled plasma atomic emission spectroscopy using ICP-AES iCAP 7400 (Thermo Scientific, Waltham, MA, USA). Metallothionein was either used directly after reconstitution or stored in aliquots at –80°C. When used after thawing, the concentration of thiolates and Zn(II) was redetermined and compared with the first determinations. After the experiments, the protein was discarded.

### Determination of the protein and metal concentration

The concentration of freshly prepared metallothionein-2 was measured using 1 mM DTNB with 1 mM EDTA in 50 mM borate buffer, pH 7.4, with 100 mM NaCl. The absorbance was measured in kinetic mode at 412 nm. The absorbance at the plateau was used for concentration calculation.^58^ Metal content was assessed using a solution containing 200 µM PAR with 1 mM DTNB in 50 mM borate buffer, pH 7.4, with 100 mM NaCl. The absorbance was measured in kinetic mode at 492 nm. The absorbance at the plateau was used for concentration calculation.^59^ Additionally, the samples were analyzed by ICP-AES iCAP 7400, Thermo Scientific to confirm the spectrophotometric results. Prior to analysis, protein samples were diluted with 0.5 M nitric acid.

### Zn(II) transfer from MT2 to ZFs

Structural changes during Zn(II) transfer from WT Zn_7_MT2 to metal-free ZF ZF133-11 and its mutant ZF133-11 C7E were monitored by CD spectroscopy (Jasco J1500 spectropolarimeter). Prior to experiments, HPLC-purified peptides were dissolved in 10 mM HCl.^60^ Samples of Zn(II)-loaded MT2 and metal-free ZF peptides were incubated in 20 mM Tris–H_2_SO_4_, pH 7.4, with 200 µM TCEP.^61^ The concentrations of peptides ZF133-11 and ZF13311 C23E remained constant at 20 µM during the experiment, while the molar ratio of Zn_7_MT2 increased from 0.1 to 2.0 relative to ZF peptides. The samples were measured in a 2 mm quartz cuvette at 25°C. Five spectra were averaged using a 5 nm band width collected at a 100 nm/min scanning speed and a 1.0 nm data pitch. Measurements were performed 3, 10, 30, and 60 min after starting the incubation in order to accurately monitor Zn(II) transfer, which changes in a time-dependent manner. Ellipticity changes were read at 222 nm. The ellipticity of the metallothionein was measured separately and later subtracted from the spectra obtained in the above-mentioned experiment.

### PTP1B inhibition by Zn(II) transferred from MT2

The commercially obtained protein tyrosine phosphatase 1B (PTP1B) was supplied in 25 mM Tris–HCl, pH 7.5, 2 mM β-mercaptoethanol, 1 mM EDTA, and 1 mM DTT. In order to remove the EDTA and thiol compounds, which could interfere with the experiment, the enzyme was purified by gel filtration on an ENrich SEC-70 column (Bio-Rad NGC). The purification was performed in 50 mM Tris–HCl, pH 7.4, with 100 µM TCEP (25°C). Fractions that exhibited strong absorbance at 280 nm were collected. Protein concentration was calculated using the Bradford method, then the concentration of the active enzyme was determined as a result of titration with Zn(II). Samples containing an equimolar amount of PTP1B and MT2 (100 nM) were incubated for 60 min in 50 mM Na^+^-HEPES, pH 7.4, with 100 mM KNO_3_ and 50 µM TCEP (25°C). In the next step, samples were added to cuvettes with 50 mM Na^+^-HEPES, pH 7.4, and 100 mM KNO_3_ and blanked.^48^ After that, para-nitrophenylphosphate (*p*NPP) substrate was added to a final concentration of 0.4 mM, which initiated the hydrolysis reaction catalyzed by PTP1B. Increase in absorbance was monitored for 2–4 min at 405 nm using a spectrophotometer. On the basis of a slope, the percentage of the activity of the enzyme was calculated. A total of 100% activity of the enzyme was calculated based on an experiment without MT2.

### Zn(II) transfer from MT2 to ZnAF-2F

Stock solution of zinc fluorescent probe, ZnAF-2F, was prepared in DMSO dimethyl sulfoxide, aliquoted, and stored at –80°C. Measurements were performed using a Horiba Fluoromax-4 spectrofluorimeter; the Peltier heating/cooling system was used to maintain the constant temperature of 25°C. The excitation/emission wavelengths were 492/515 nm.^62^ Two sets of 1 cm cuvettes were filled with 50 mM Na^+^-HEPES pH 7.4, 100 mM NaClO_4_, and 100 µM TCEP. Fluorescent probes were added in the concentrations of 0.05–2.0 µM, and initial fluorescence (F_0_) was measured. Then, for one set, MT2 (fully loaded) was added to a final concentration of 0.5 µM and incubated for 2 h, and fluorescence F was recorded. During the incubation, cuvettes were closed with parafilm and protected from light. After the measurement, the readout was calibrated by the addition of ZnSO_4_ to a final concentration of 40 µM (F_max_), followed by EDTA to a final concentration of 0.3 M (F_min_). Control MT2 samples contained 5 µM of the ZnAF-2F probe, and 20 µl of 1 mM DTNB was added for total thiolates’ oxidation and Zn(II) release.

### Zn(II) transfer from MT2 to PAR

The experiments were performed in 50 mM Na^+^-HEPES buffer, pH 7.4, with 0.1 M NaCl treated with Chelex 100 (Bio-Rad) and 200 µM TCEP (25°C). The 20 mM PAR stock solution was prepared freshly in anhydrous DMSO.^59^ The experiments were performed with the reference cuvette in kinetic mode on a double-beam Jasco V-650 spectrophotometer, and data points were collected at 492 nm every 5 s. In order to study the relation between the percentage of Zn(II) transferred to PAR, probe stock solution was added to the samples to its final concentration from 2 to 400 µM. Higher concentrations resulted in probe or Zn(II) complex precipitation under the used conditions. After 1 min from the start, MT2 was added to a final concentration of 1.7 µM, and the absorption was monitored for ∼50 min; however, 30 min was enough for the equilibration in all cases.

### Zn(II) transfer from MT2 to ATP, L-His and GSH by SEC

Zn(II) transfer from MT2 to weakly bound ligands was analyzed by size exclusion chromatography (SEC) in 50 mM Na^+^-HEPES buffer, pH 7.4, containing 100 mM NaClO_4_ (25°C), using an ENrich-SEC 70 column (Bio-Rad) on FPLC (Bio-Rad NGC). Stock solutions of glutathione (GSH), L-histidine, and adenosine triphosphate (ATP) at a concentration of 250 mM were prepared in Milli-Q water just prior to the experiments, and their pH was adjusted to 7.4 using metal-free concentrated NaOH. Incubation of MT2 with ligands was performed in running buffer (total sample volume 200 µl) in such a way that the fully Zn(II)-loaded MT2 concentration was 38 µM MT2 (fully loaded) while the ligand concentration was either 20 mM or 40 mM. After incubation at 25°C for 1 h, samples were centrifuged and loaded onto an FPLC column, and separation was run for 30 min with 1 ml/min buffer flow. A control sample of Zn_7_MT2 without any ligand was also prepared and analyzed. The fraction containing eluted MT2 (1 ml) was collected and assayed for total Zn(II) and thiol concentrations using PAR and DTNB assays, respectively, as described above. The measurements were performed in four separate repetitions.

### MT2 and ZF domain competition monitored by ESI-MS

The competition for Zn(II) between human MT2 and ZF peptides ZScan20, ZNF442, and CP1 (CCHH) was semi-quantitatively examined by ESI-MS. All samples containing 30 µM apo-MT2 and metal-free peptides were prepared freshly in degassed 100 mM ammonium acetate, pH ∼7.5. To those mixtures, ZnSO_4_ was added, so the final molar ratio of Zn(II)-to-MT2 between 5 and 8. The mixtures were incubated for 10 min at room temperature before injection into the mass spectrometer. Mass spectra were collected on a Bruker Compact QTOF (Bruker Daltonics) operated in the positive ionization mode and calibrated with ESI Tuning Mix (Agilent Technologies). Sample solutions were introduced into the MS at a rate of 3 µl/min. Settings: scan range = 400–3000 m/z, nebulizer = 1.8 bar, dry gas = 220°C and 8.0 l/min, capillary = 4500 V, end plate offset = 500 V, and hexapole RF = 300 Vpp. The spectra were averaged >1–2 min collection time and deconvoluted using the maximum entropy mode of the Bruker Compass Data Analysis software package. The deconvoluted signal intensity of the holo and apo forms of a particular ZF was used to calculate the percentage of the two species in the sample.

## Results and discussion

### Production of zinc MT2 in bacterial system and its initial characterization

MTs are highly heterogeneous proteins due to their presence in various (sub)isoforms, tendency for oxidation or binding various essential and toxic metal ions, reactivity, and posttranslational modifications.^1,2,63–65^ Currently, the most common procedure for obtaining MTs is the production of recombinant proteins in the bacterial system.^54,66–69^ This method makes it possible to obtain a certain isoform of metallothionein in its chemically pure form. However, depending on the used expression system and vectors obtained, MTs may contain additional amino acid flanks either from the N- or C-terminal tail. In this study, recombinant human MT2 (also known as MT2a or MT-II) was produced using the IMPACT system. It allows self-cleavage of the fusion protein during purification, which is produced without any flank on the target protein.^27,54^ MT2 was chosen because it is the most explored isoform regarding metal-binding properties, structure, reactivity, etc.^2^

**Fig. 2:**
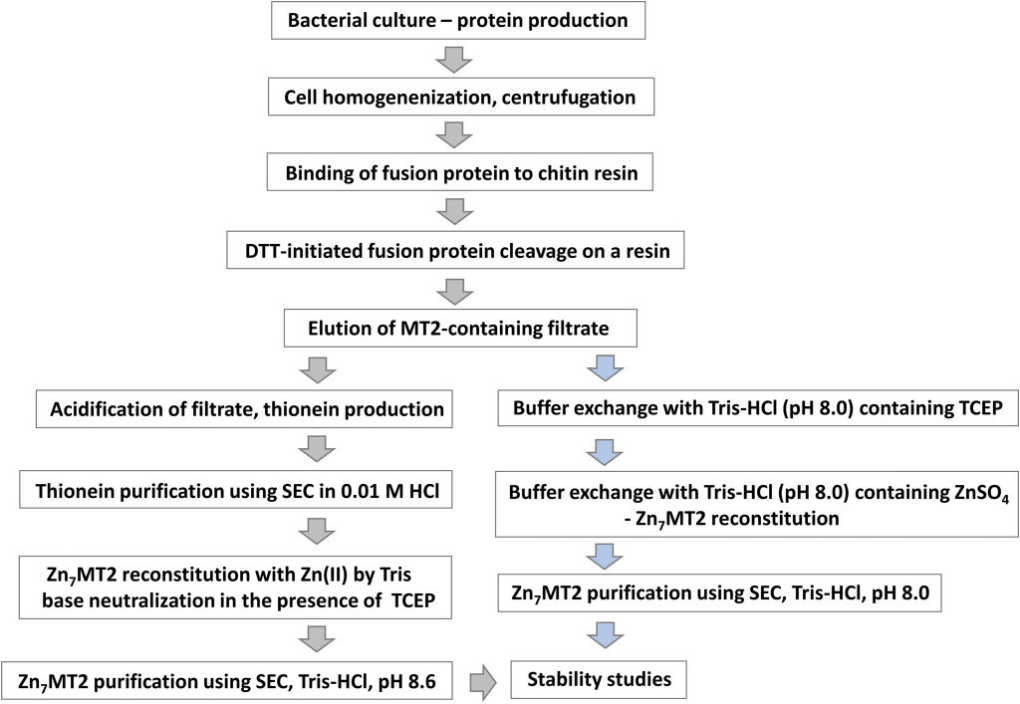
Workflow of recombinant human MT2 production performed in this study. Zn(II)-loaded MT2 was obtained in two approaches: initial thionein (metal-free form) purification at pH ∼2 followed by metal reconstitution (left panel) and Zn(II) loading at pH 8.0 without initial acidification (right panel). Buffer exchange was performed using centrifugal filters.

Figure 2 shows the workflow for the preparation of homogeneous zinc MT2 used in this study, as described in detail in the methods. The DTT has a double function here. It cleaves the intein and additionally protects thiols and thiolates of MT against air oxidation since it has a lower redox potential than β-mercaptoethanol, resulting in higher efficiency.^70^ It should be underlined that the actual redox status of MTs cleaved from resin-attached fusion is unknown; however, the applied procedure was shown to be efficient in full protein reduction.^54^ As it has been previously shown that major acidification can affect MT2 Zn(II)-binding characteristics, we performed the purification and Zn(II) loading in two different approaches. According to the first one, the elute was subsequently acidified to pH ∼2 using 1 M HCl (left panel of Fig. 2), which causes metal dissociation from MT2 bound in bacteria and additionally protects proteins against oxidation due to thiolates’ protonation. Acidified protein was then separated by SEC using 10 mM HCl as a mobile phase, which allowed its separation from DTT and other impurities. Freshly collected metal-free MT2 (thionein) was mixed with a low excess of ZnSO_4_ [∼8 Zn(II) mol. eq.] in the presence of TCEP, and then pH was increased by Tris base to initiate Zn(II) binding. TCEP was used here since it is a reducing agent that very weakly binds Zn(II), in contrast to DTT, which binds Zn(II) efficiently and may affect the final protein composition.^61,71^ Finally, pure Zn(II)-loaded MT2 was obtained by SEC separation at pH 8.6, which additionally protected Zn(II) dissociation from MT2. On average, ∼2.1 mg of high quality Zn(II)-loaded MT2 was produced from one liter of bacterial culture. To monitor the amount of protein during expression or preparation and avoid faint staining of the MT in SDS-PAGE gels by Coomassie dye, we applied the biarsenical fluorescent probe F4FlAsH-EDT_2_.^56^ This probe, almost non-fluorescent in the absence of MT, became fluorescent in the presence of MT and EDTA due to its covalent attachment to Cys residues of MT2.^72,73^

Due to some suggestions present in the literature that MT acidification during its purification may damage the protein or change its metal-binding properties, Zn(II)-loaded MT2 was obtained in the route without acidifying the recombinant protein (right panel of Fig. 2).^40^ For that purpose, cleaved MT2 with DTT was first washed with metal-free Tris–HCl buffer (pH 8.0) with TCEP and then ZnSO_4_ was added to the TCEP-containing Tris–HCl buffer. In this way, the majority of DTT and EDTA was removed and the protein was saturated with Zn(II). To obtain TCEP-free Zn(II)-loaded MT2, the protein was purified in gel filtration.

MT2 prepared in two different approaches was assayed with DTNB in the presence of EDTA to determine the concentration of thiolates. Since the purification, excluding the last step, was performed in the presence of TCEP, we assumed all thiols were reduced. Then, Zn(II) concentration was determined with PAR in the presence of DTNB as the oxidation agent.^55^ In all measurements, the determined Zn(II)/thiol(ate) concentration ratio varied from 0.335 to 0.352 regardless of the procedure whereby it was obtained. It corresponds to the expected Zn(II)/protein ratio of 6.7−7.0. In the second step, the same samples were subjected to ICP-AES measurements to independently determine zinc and sulfur concentrations. In that case, the Zn(II)/protein ratio in all measurements varied from 6.8 to 7.3, indicating that the experimental error was not >0.3 of Zn(II) mol. eq. equivalent over protein. The obtained results indicate that recombinant MT2 is fully loaded and reduced, regardless of the used procedure (Fig. 2), and acidification does not affect the metal loading characteristics.

### Determination of Zn(II) dissociation constant of Zn(II)-MT2 by the competition with PAR

Among all available Zn(II)-sensitive probes, PAR is the most applicable due to its ease of use and low price compared to fluorescent counterparts. It demonstrates very good spectral properties (ε_492_ = 71 500 M^−1^ × cm^−1^ at pH 7.4) and medium Zn(II) affinity with *K*_12_^PAR^ = 1.4 × 10^12^ M^−2^. However, due to the formation of predominant 1:2 complexes [ZnH_x_(PAR)_2_] it is sometimes omitted in favor of Zincon (ZI), which binds Zn(II) with 1:1 stoichiometry (*K*^ZI^ = 4.8 × 10^5^ M^−1^), but demonstrates a significantly lower signal boost upon Zn(II) binding at pH 7.4 (ε_618_ = 24 200 M^−1^ × cm^−1^).^59,74^ PAR has been used in numerous studies with various MTs either to monitor Zn(II) dissociation (e.g. oxidation or alkylation of Cys residues or main-chain hydrolysis) and its binding or to investigate Zn(II)-binding affinity to the MTs or MT-like proteins.^44–46,75–78^ Due to the fact that thermodynamic data obtained for PAR competition with mammalian MTs differ significantly between each other, in this study, we aimed to verify its usefulness for the determination of Zn(II) in human MT2, with special attention to the fact that not all metal ions are bound with the same and high affinity by the protein.

**Fig. 3:**
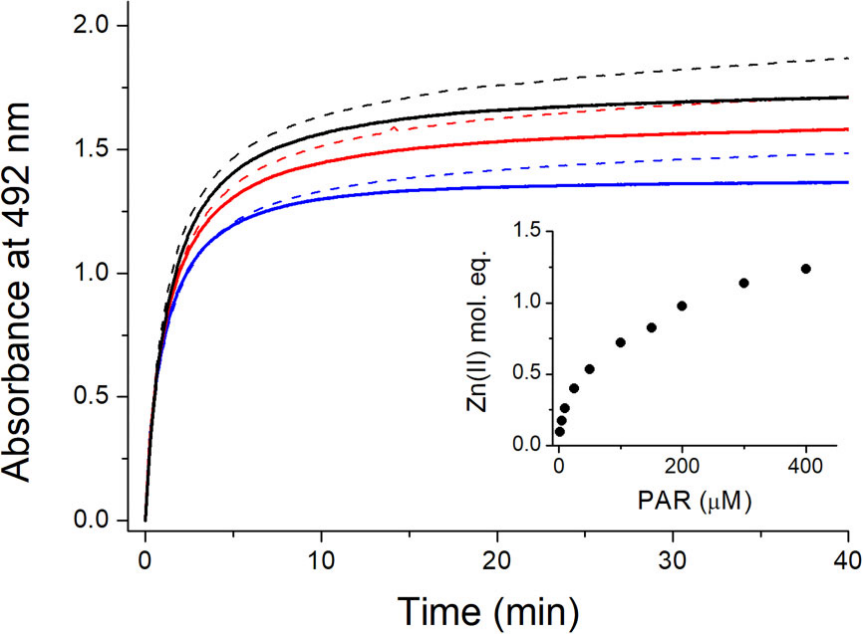
Kinetics of Zn(II) transfer from MT2 (1.7 µM) to 200 (blue), 300 (red), and 400 µM PAR (black) in 50 mM Na^+^-HEPES buffer, pH 7.4, *I* = 0.1 M, 25°C. Solid and dashed lines show kinetics of Zn(II) transfer in the presence and absence of 200 µM TCEP as the reducing agent. Inset demonstrates the relationship between molar equivalents of transferred Zn(II) and total concentration of PAR in the presence of TCEP.

The competition between fully Zn(II)-loaded MT2 (Zn_7_MT2) and PAR was performed in such a way that MT2 concentration was constant in all measurements (1.7 µM) and PAR varied from 5 to 400 µM, which corresponds to a molar excess over MT2 from 2.9 to 235. Figure 3 shows an example of kinetics of Zn(II) transfer from Zn_7_MT2 to 200, 300, and 400 µM PAR in the presence of TCEP and its absence. In all samples with TCEP, a characteristic plateau was observed after 30 min, while no absorbance change was noted above ∼60 min. The lack of TCEP resulted in a continuous absorbance increase at 492 nm above 30 or even 60 min due to the oxidation of cysteinyl residues. In such a situation, the transfer of the weakest Zn(II) accelerates the dissociation of other ions, which become less tight in partially oxidized protein.^79^ Such continuous kinetics have been observed in previous studies where TCEP or another reducing agent was not added to the sample.^47,80^ This observation proves that TCEP is required for any kind of interaction of MTs with metal chelators because it warranties proper equilibration and a reduced state.^61^ To obtain quantitative information on how many Zn(II) mol. eq. were transferred to PAR, absorbance at 492 nm was converted to Zn(PAR)_2_ (the protonation state of PAR molecules is omitted later for clarity) concentration using the molar absorption coefficient. The inset of Fig. 3 shows the relation between transferred Zn(II) and total PAR concentration. Up to 150 µM of PAR, <1 Zn(II) mol. eq. from MT2 is transferred. Above that concentration, starting from 200 µM, >1 Zn(II) mol. eq. is transferred. For instance, at 400 µM PAR, ∼1.2 mol. eq. of Zn(II) is transferred from MT2. Practically, it means that PAR is able to compete not only with the weakest bound Zn(II) in Zn_7_MT2 (Equation 1) but also with the second weakest Zn(II) of Zn_7_MT2 (actually the weakest of Zn_6_MT2) species (Equation 2). Exchange constant *K*_ex_ that relates only Zn_7_MT2 and Zn_6_MT2 is defined by Equation (3). It is worth noting that it is not easy to determine successive (step) dissociation constants of Zn_7_MT2 such as *K*_d1_ (Equation 4), *K*_d2_, etc., from this kind of experiment since particular events overlap each other and knowledge about the whole system would be required, which is not the case. However, by combining Equations (3) and (4), one is able to calculate *K*_d1_ from the initial competition points, which are weakly affected by the second Zn(II) dissociation event (Equation 5).


(1) \begin{equation*}{\mathrm{Z}}{{\mathrm{n}}}_7{\mathrm{MT}}2 + 2{\mathrm{PAR}} \rightleftharpoons {\mathrm{Z}}{{\mathrm{n}}}_6{\mathrm{MT}}2 + {\mathrm{Zn}}{\left( {{\mathrm{PAR}}} \right)}_2,\end{equation*}

(2) \begin{equation*}{\mathrm{Z}}{{\mathrm{n}}}_6{\mathrm{MT}}2 + 2{\mathrm{PAR}} \rightleftharpoons {\mathrm{Z}}{{\mathrm{n}}}_5{\mathrm{MT}}2 + {\mathrm{Zn}}{\left( {{\mathrm{PAR}}} \right)}_2,\end{equation*}

(3) \begin{equation*}{K}_{{\mathrm{ex}}} = \frac{{\left[ {{\mathrm{Z}}{{\mathrm{n}}}_6{\mathrm{MT}}2} \right] \cdot \left[ {{\mathrm{Zn}}{{\left( {{\mathrm{PAR}}} \right)}}_2} \right]}}{{\left[ {{\mathrm{Z}}{{\mathrm{n}}}_7{\mathrm{MT}}2} \right] \cdot {{\left[ {{\mathrm{PAR}}} \right]}}^2}}\ ,\end{equation*}

(4) \begin{equation*}{K}_{d1} = \frac{{\left[ {{\mathrm{Z}}{{\mathrm{n}}}_6{\mathrm{MT}}2} \right] \cdot {{[{\mathrm{Zn}}\!\left( {{\mathrm{II}}} \right)]}}_{{\mathrm{free}}}}}{{\left[ {{\mathrm{Z}}{{\mathrm{n}}}_7{\mathrm{MT}}2} \right]}}\ ,\end{equation*}

(5) \begin{equation*}{K}_{{\mathrm{d}}1} = {K}_{{\mathrm{d}}12}{\!}^{{\mathrm{PAR}}} \times {K}_{{\mathrm{ex}}}.\end{equation*}

**Fig. 4:**
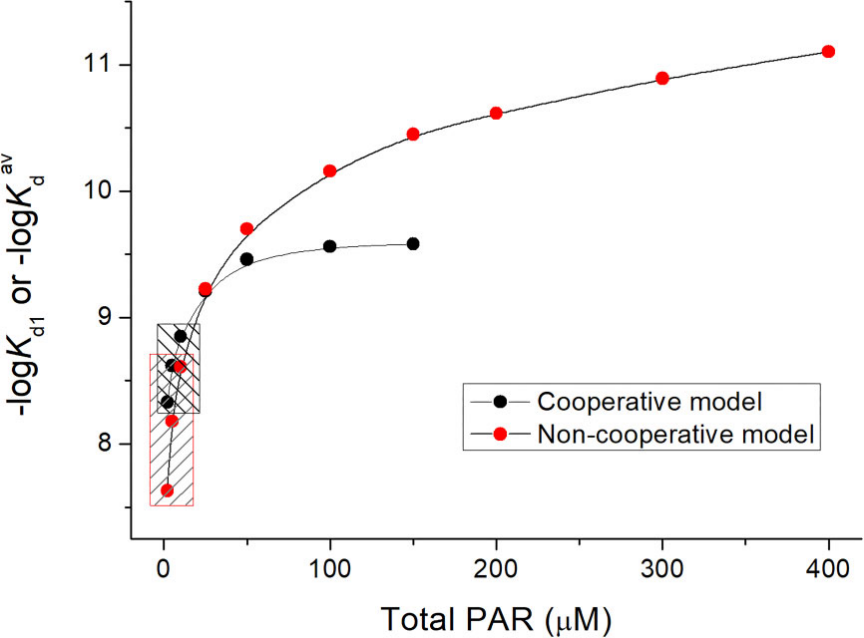
Relation between the determined first-step dissociation constant (*K*_d1_, non-cooperative model) or average dissociation constant (*K*_d_^av^, cooperative model) and total PAR concentrations. Black and red boxes indicate experimental points averaged in this experiment (see Fig. 3). The competition was performed using 1.7 µM MT2 (Zn_7_MT2) in 50 mM Na^+^-HEPES, pH 7.4, 200 µM TCEP, *I* = 0.1 M, 25°C.

Figure 4 shows relations between calculated –log*K*_d1_ and total PAR concentration. Calculated *K*_d1_ values from experimental points (Table S3) are not constant and vary from 7.6 to 9.6; therefore, for the calculation of *K*_d1_, only the first three points were taken into consideration (black box of Fig. 4). It results in a value of 8.6 ± 0.3 (Table 2), which is significantly weaker than the average *K*_d_^av^ value (average affinity of all metal ions; see below) determined by the competition with other ligands (Table S3).^38,40,41,45^ However, it matches our previous observations that the weakest Zn(II) is bound to MT2 with nanomolar affinity.^32^

**Table 2.** Competitors and dissociation constant of the weakly bound Zn(II) in MT2 in competition experiments performed in this study. All constants were determined at pH 7.4 (excluding ESI-MS) in various buffers specified in *Experimental procedures*

Competitor	Method of constant(s) determination	–log*K*_d_ of Zn(II)-competitor complex (*K*_d_ value)	Reference	–log*K*_d1_^a^ of weakest zinc site in MT2
PTP1B	Kinetics (UV-vis)	7.8 (1.6 × 10^−8 ^M)	[48]	8.2 ± 0.2
PAR	Spectroscopic (UV-vis)	12.1 (7.1 × 10^−13^ M^2^)	[59]	8.6 ± 0.3 8.1 ± 0.5
ZF133-11	Spectropolarimetric (CD)	12.5 (3.31 × 10^−13^ M)	[59]	Too weak to be determined^b^
ZF133-11_C/E_	Spectropolarimetric (CD)	8.4 (3.63 × 10^−9^ M)	[59]	8.6 ± 0.1
ZnAF-2F	Spectrofluorimetric (FL)	8.3 (5.5 × 10^−9^ M)	[62]	8.32 ± 0.01 8.19 ± 0.01^c^
ATP	Size exclusion chromatography (SEC)	5.1 (7.7 × 10^−6^ M)	[81]	7.9 ± 0.2^e^
GSH	Size exclusion chromatography (SEC)	6.4–6.7^d^ (2 × 10^−7^ – 4 × 10^−7^ M)	[82]	8.9 ± 0.1^e^
L-His	Size exclusion chromatography (SEC)	6.8–7.2^d^ (1.6 × 10^−7^–6.3 × 10^−8^ M)	[82]	8.7 ± 0.3^e^
Average *–*log*K*_d1_^a^ of weakest zinc site in MT2 from all solution-based experiments				8.4
ZScan20	Mass spectrometry (ESI-MS)	7.9 (1.3 × 10^−8^ M)	[76]	∼11
ZNF442	Mass spectrometry (ESI-MS)	10.4 (4.0 × 10^−11^ M)	[76]	∼11
CP1-2015 (CCHH)	Mass spectrometry (ESI-MS)	12.3 (5.0 × 10^−13^ M)	[60]	Too weak to be determined^b^

^a^The *K*_d_ of single (weakest) binding site.

^b^Values were not determined due to the large difference in the affinity of the competitor and weakly bound Zn(II) site in MT2.

^c^Dissociation constant of the weakly bound Zn(II) in MT2 reconstituted without protein acidification (see Experimental procedures).

^d^Presented values are *CI. CI* is the logarithm of the apparent dissociation constant of the ZnZ complex [Zn(II) complex of theoretical molecule Z], such as (ZnZ) =  Σ_ijk_(Zn_i_H_j_L_k_) at given overall component concentrations. Total concentrations of Z were set to either 20 or 40 mM, and Zn(II) concentration bound to Z was determined after SEC. Note that Zn_i_H_j_L_k_ concentrations were calculated using protonation and stability constants of His, ATP, and GSH using Hyperquad software.^82,93,98^

^e^ *K*_d1_, defined by Equation (1), was calculated using total concentrations of MT2 and competing ligands by Hyperquad software.^93^ Zn(II) bound to competing ligand and Zn_7_MT2 and Zn_6_MT2 concentrations was determined after reactants separation using SEC.

The above-presented data analysis of chromogenic zinc chelator competition with MT2 considering Zn(II) step dissociation processes is not widely described in the literature, with just a few exceptions.^32,44^ Instead, commonly, to determine Zn(II) affinity for a particular MT protein, a significant simplification of the system is used, according to which MT is treated as a molecule that binds all Zn(II) ions [in the case of mammalian MT2, all seven Zn(II) ions] with the same, not distinguished, affinity. Therefore, the exchange equilibrium between PAR and Zn_7_MT2 (Equation 6) is then presented differently than in Equations (1) or (2). In that case, all Zn(II) ions bound to MT ions are mobilized during equilibration. The new exchange constant *K*_ex_^coop^ (Equation 7) relates therefore fully metallated Zn_7_MT2 and metal-free MT2 in a cooperative manner.


(6) \begin{equation*}{\mathrm{Z}}{{\mathrm{n}}}_7{\mathrm{MT}}2 + 14{\mathrm{PAR}} \rightleftharpoons 7{\mathrm{Zn}}{\left({\mathrm{PAR}} \right)}_2 + {\mathrm{MT}}2,\end{equation*}

(7) \begin{equation*}K_{{\mathrm{ex}}}{\!}^{{\mathrm{coop}}} = \frac{{{{[{\mathrm{Zn}}{{\left( {{\mathrm{PAR}}} \right)}}_2]}}^7 \cdot \left[ {{\mathrm{MT}}2} \right]}}{{\left[ {{\mathrm{Z}}{{\mathrm{n}}}_7{\mathrm{MT}}2} \right] \cdot {{\left[ {{\mathrm{PAR}}} \right]}}^{14}}}\ .\end{equation*}

In order to calculate the cooperative dissociation constant *K*_d17_^av^ of Zn_7_MT2 (Equation 8) by analogy to the step-wise dissociation mechanism (see above), *K*_ex_^coop^ and *K*_d12_^PAR^ must be combined in one equation, Equation (9). All numerical details of the calculations are presented in Table S4.


(8) \begin{equation*}K_{{\mathrm{d}}17}{\!}^{av} = \frac{{{{[{\mathrm{Zn}}\left( {{\mathrm{II}}} \right)]}}^7 \cdot \left[ {{\mathrm{MT}}2} \right]}}{{\left[ {{\mathrm{Z}}{{\mathrm{n}}}_7{\mathrm{MT}}2} \right]}}\ ,\end{equation*}

(9) \begin{equation*}{K}_{{\mathrm{d}}17}{\!}^{{\mathrm{av}}} = {K}_{\mathrm{d}}{\!}^{{\mathrm{PAR}}} \times {K}_{{\mathrm{ex}}}{\!}^{{\mathrm{coop}}}.\end{equation*}

Because *K*_d17_^av^, due to its definition, is provided in the M^7^ unit and its value varies from 10^−54^ to 10^−78^ M^7^ (Table S4), it combines all step constants from *K*_d1_ to *K*_d7_ that are equal to each other according to the adopted assumption (*K*_d1_ = *K*_d2_ = … = *K*_d7_). To calculate the average *K*_d_^av^ valid for one bound Zn(II) ion, this value is simply divided by seven (Equation 10).


(10) \begin{equation*}{K}_{\mathrm{d}}{\!}^{{\mathrm{av}}} = {K}_{{\mathrm{d}}17}{\!}^{{\mathrm{av}}}/7.\end{equation*}

The obtained –log*K*_d_^av^ value presented in Fig. 4 varies significantly from the lowest (7.63) to the highest (11.1) PAR concentration used in the competition, differently from the non-cooperative model. For instance, the *K*_d_^av^ value determined here at the PAR concentration of 200 µM is 2.4 × 10^−11^ M and is almost identical to 3.1 × 10^−11^ M, determined recently by Calvo *et al.* for the same protein under very similar conditions.^45^ The significant increase of *K*_d_^av^ at higher PAR concentrations indicates that under those conditions not only the first but also the second or even the third Zn(II) ion is mobilized from MT2. Therefore, *K*_d_^av^ indeed reflects an average value. If the application of PAR were possible at even higher concentrations than in the experiment, then a higher –log*K*_d_^av^ would probably be obtained and also would represent the averaged affinity of the tightest Zn(II) ions. Therefore, *K*_d_^av^ points taken from the lowest used PAR concentration are much less influenced by the second or the third Zn(II) dissociation event and represent mostly the first one (∼*K*_d1_), indicating nanomolar Zn(II) affinity (Table 2).

### Determination of Zn(II) dissociation constant of Zn(II)-MT2 by competition with fluorescent probe ZnAF-2F

The observation that different PAR concentrations result in different determined dissociation constants indicated that Zn(II)-binding sites with different affinities are mobilized to varying degrees in competition with a ligand of moderate Zn(II) affinity. Since the mobilization of one Zn(II) mol. eq. from MT2 requires ∼150 µM PAR, a large free PAR excess over MT additionally increases the chance of non-specific interaction with proteins present at a hundred-fold lower concentration. It is worth examining whether the same conclusion derives from the competition of Zn_7_MT2 with a zinc probe with slightly higher (but still moderate) Zn(II) affinity and high sensitivity toward Zn(II). The obvious candidates for such a type of test are fluorescent zinc probes, which demonstrate a large signal increase upon Zn(II) binding over the metal-free form. From commercially available probes, only a few can be taken into account. Since FluoZin-3 and RhodZin-3 were used in previous studies, here we used ZnAF-2F and investigated its interaction with MT2 to observe how it differs from MT2 obtained alternatively (MT2^pH8^) to the standard protocol, excluding protein acidification during the reconstitution process. Another practical advantage of ZnAF-2F is the formation of only a 1:1 Zn(II) complex at neutral pH, in contrast to PAR, which under certain conditions may form ZnPAR and Zn(PAR)_2_ complexes at the same time.^59,62^

To study Zn(II) transfer to ZnAF-2F, 0.5 µM metal-saturated MT2 was incubated with increased concentration of the probe starting from 0.05 to 5 µM in the presence of TCEP for 2 h. It corresponds to only a 0.1–10 molar ratio over protein, which is ∼40 times less than PAR. Fluorescence (F) of all samples of both protein preparations, MT2 and MT2^pH8^, was measured after 2 h of incubation. To calculate how much Zn(II) was transferred to the probe, the samples required calibration. For that purpose, first to the samples, after recording F values, an excess of ZnSO_4_ was added to saturate the probe with Zn(II) (F_max_), and then the excess of EDTA to reach minimal fluorescence (F_min_) was supplemented. The final determination of transferred Zn(II) equivalents was performed by the comparison of F values to the fluorescence measured by MT2 oxidation under the same conditions (without TCEP) in the presence of DTNB and 5 µM ZnAF-2F. To determine dissociation of the weakest zinc site in MT2, first all recorded intensities (F, F_min_, F_max_) were converted to the metal-free probe (ZnAF-2F) and its Zn(II) complex (Zn-ZnAF-2F) molar concentrations according to Equation (11).


(11) \begin{equation*}K_{\mathrm{d}}^{{\mathrm{ZnAF}}\hbox{-}2{\mathrm{F}}} = \frac{{{{[{\mathrm{Zn}}({{\mathrm{II}}})]}}_{{\mathrm{free}}}\left[ {{\mathrm{ZnAF\hbox{-}2F}}} \right]}}{\left[{{\mathrm{Zn}}({{\mathrm{II}}})\hbox{-}{\mathrm{ZnAF}}\hbox{-}2{\mathrm{F}}} \right]}\ = \frac{{\left[ {{\mathrm{Zn}}\!\left( {{\mathrm{II}}} \right){]}_{{\mathrm{free}}}} \right[{\mathrm{F}} - {{\mathrm{F}}}_{{\mathrm{min}}}]}}{{\left[ {{{\mathrm{F}}}_{{\mathrm{max}}} - {\mathrm{F}}} \right]}}\ ,\end{equation*}

(12) \begin{equation*}{[{\mathrm{Zn}}({\mathrm{II}})]}_{{\mathrm{free}}} = K_{\mathrm{d}}^{({\mathrm{ZnAF}}\hbox{-}2{\mathrm{F}})} \cdot \frac{{[{{\mathrm{F}}}_{\max } - {\mathrm{F}}]}}{{[{\mathrm{F}} - {{\mathrm{F}}}_{\min }]}}.\end{equation*}

**Fig. 5:**
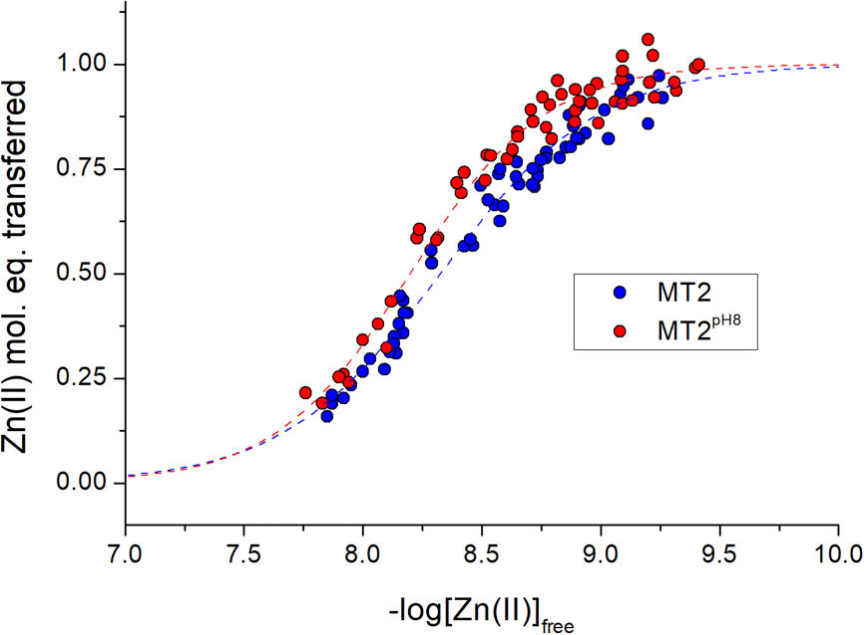
Determination of the dissociation constant of weak binding site in MT2 obtained after acidification (blue) and directly at pH 8 (red) by the quantification of Zn(II) transfer from the protein to ZnAF-2F. Protein (0.5 µM) was incubated with 0.05 to 5 µM probe >2 h in 50 mM Na^+^-HEPES buffer (pH 7.4, 200 µM TCEP, *I* = 0.1 M, 25°C) prior to fluorescence measurements. Measurements were performed in triplicate.

Then free Zn(II) concentrations present at each sample of MT2 with various ZnAF-2F concentrations after equilibration were calculated based on measured F, F_min_, and F_max_ intensities according to Equation (12). These values were used to correlate them with the number of Zn(II) mol. eq. transferred to ZnAF-2F during the competition (Fig. 5). It shows that regardless of the acidification of MT2 during preparation, the profile of Zn(II) transfer is highly similar. Within the used ZnAF-2F, the concentration transfer of ∼1 Zn(II) mol. eq. is observed. It means that the probe was able to compete with the weakest bound Zn(II) ion. Inflection points of these isotherms correspond to –log*K*_d_ values of the weakest Zn(II)-binding site, 8.3 and 8.1 for proteins obtained with and without acidification during holo-form preparation, respectively (Table 2). At this point, it is not clear how the second Zn(II)-binding site contributes to the observable metal transfer and how the obtained constant is affected by that process. However, Hill's coefficients (n), 1.3 and 1.5 for MT and MT^pH8^, may suggest that the observed 1 Zn(II) mol. eq. could be transferred not only from the weakest but partially from the second weakest Zn(II)-binding site in MT2. Figure 5 indicates that the plateau above –log[Zn(II)]_free_ ∼9 is not clearly defined and Zn(II) transfer could continue at a higher probe concentration as in the case of PAR. However, use of a higher ZnAF-2F concentration was not possible due to probe precipitation and a non-linear fluorescence response. What is clear here is the fact that the weakest Zn(II) is transferred under nanomolar free Zn(II) concentration, similarly to PAR and previously used FluoZin-3 and RhodZin-3.^32^ The significantly lower concentration of ZnAF-2F than PAR used in this study minimizes the chance for non-specific interaction with protein; however, the formation of ternary complexes cannot be excluded without additional investigation.^83–85^ Slight differences in isotherms presented in Fig. 5 (both –log*K*_d1_ and slope values) may also come from different pH (8.6 vs. 8.0) applied during Zn(II)-reconstitution. It should be emphasized that the stability of tetrathiolate zinc sites [Zn(Cys)_4_] and, therefore, zinc saturation status depends on pH.^2^ Protein reconstituted at a lower pH may contain a lower amount of Zn(II) than that obtained at a higher value. Results from this study show comparable Zn(II) saturation (see above); however, a slight difference, lower than an experimental error of spectroscopic assays or ICP-AES analysis, still might be possible. From the other hand, the amount of Zn(II) mol. eq. transferred to ZnAF-2F is identical for both proteins, proving their high similarities regarding composition and properties.

### MT2 competition with ZF domains

Although chromogenic and fluorogenic probes are commonly used to monitor (free) metal concentrations and report competition with metal-binding proteins, we also explored other alternatives for competition-based experiments.^59,74,86,87^ In this study, we aimed to investigate how zinc MT2 interacts with ZF domains common in biology—structural zinc sites of numerous proteins involved in DNA and RNA processing.^88–90^ It has been reported that ββα ZFs bind Zn(II) very tightly with picomolar and femtomolar affinities in order to serve structural functions regardless of Zn(II) fluctuations.^60,88,91,92^ However, recent studies have shown that certain ZF motifs contain partially altered metal-binding sites and bind Zn(II) with nanomolar affinity, forming functional structures. These ZFs seem to be transiently saturated under cellular conditions and may participate in important Zn(II)-dependent cellular processes.^76^ Here, we used two types of ZFs: one with low-subpicomolar affinity, ZF133-11 (–log*K*_d_ = 12.5), and its altered version ZF133-11_C/E_ demonstrating four orders of magnitude weaker Zn(II)-binding affinity (–log*K*_d1_ = 8.4).^52,59^

**Fig. 6:**
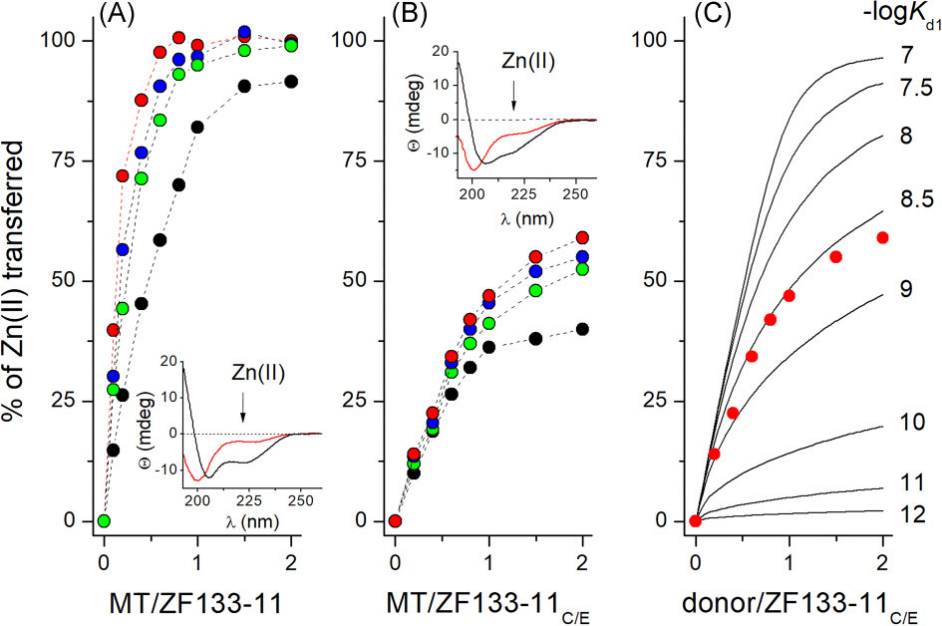
Zn(II) transfer between MT2 and metal-free ZFs. (A) Saturation of 5 µM ZF133-11 by MT2 at 0–2 MT/ZF molar ratios monitored by ellipticity changes at 222 nm. Black, green, blue, and red circles represent fractions of ZF saturation after 3, 10, 20, and 60 min of equilibration. (B) Saturation of 5 µM ZF133-11_C/E_ by MT2 at similar conditions as above. Insets of (A) and (B) show CD spectra of metal-free (red) and Zn(II)-saturated (black) ZFs. Arrows demonstrate ellipticity changes monitored at 222 nm upon Zn(II) binding. (C) Simulation of Zn(II) transfer to 5 µM ZF133-11_C/E_ from a hypothetical zinc site with –log*K*_d1_ varying from 7 to 12. Red circles correspond to experimental data recorded after 60 min of equilibration.

At the first stage of this study, both ZF peptides were titrated with ZnSO_4_ and their folding was monitored by CD spectroscopy (Fig. S1). Regardless of the difference in the coordination sphere and shape of CD spectra, the observable effect upon Zn(II) binding remains comparable for both motifs. Next, Zn(II) transfer from MT2 to metal-free ZFs was performed by 5 µM ZF titration with 0–10 µM MT2. Ellipticity changes were monitored at 222 nm and are presented in Fig. 6A (ZF133-11) and Fig. 6B (ZF133-11_C/E_). Due to the time dependence of this process, the whole Zn(II) transfer was monitored after 3, 10, 20, and 60 min. The results show that ZF133-11 was fully saturated by Zn(II) at ∼1 molar MT2/ZF ratio almost immediately after mixing of reactants. However, at lower MT2/ZF ratios, equilibration took more time, indicating full ZF saturation at ∼0.5 MT2/ZF ratio after 1 h. It means that the weakest Zn(II) from MT2 is mobilized immediately, while the second is transferred more slowly. It informs us also that not only the first, but at least the second weakest Zn(II) site of Zn_7_MT2 is bound to MT2 with affinity that is weaker than in the case of ZF133-11. A similar observation was made before for apo-SDH titrated with Zn(II)-loaded MT1 or MT2.^47,48^ Zn(II) transfer to ZF133-11_C/E_ is significantly different. In this case, the full saturation of ZF is not reached even at MT2 excess; moreover, the shape of the saturation curves suggests that only one site from MT2 (the weakest one) contributes to ZF loading, opposite to ZF133-11 (Fig. 6B). Such an observation can be explained by the transfer of the weakly bound Zn(II) with nanomolar affinity due to comparable stability of ZFs. It stems also from the fact that ZF133-11C/E is only ∼60% saturated at a 2.0 MT2/ZF molar ratio. To obtain information on the stability of the weakest site of MT2, the ZF saturation profile was compared with simulated curves of a hypothetical zinc site with –log*K*_d_ varying from 7 to 12. Simulations were performed using HySS speciation software.^94^ As can be seen in Fig. 6C, the experimental data show a good overlap with the simulation curve plotted for –log*K*_d_ = 8.6 ± 0.1 (Table 2). Obviously, we are unable to conclude what fraction of Zn(II) from the second weakest zinc site could contribute to that process; therefore, we limit discussion to the first one. Thus, the stability constant of that site is convergent with data obtained from zinc probes’ competition (Table 2).

### Inhibition of PTP1B by MT2 due to Zn(II) transfer

The above-presented data on the different mechanisms of ZFs’ saturation by MT2 depending on their affinity for Zn(II) strongly suggest that such regulation is present for structural sites. However, it is not limited to such sites. Previous studies have shown that various apo- or Zn(II)-regulated enzymes can be activated by zinc metallothioneins, but this process was not fully understandable due to the common misinterpretation that mammalian zinc MTs bind all seven Zn(II) ions with the same low-picomolar affinity.^46,47,94–96^ Here, by the use of PTP1B, whose activity is selectively quenched by Zn(II) binding with nanomolar affinity (*K*_d_ = 1.6 × 10^−8 ^M), we show how Zn(II) affinity of MT2 can be investigated.^48–97^

**Fig. 7:**
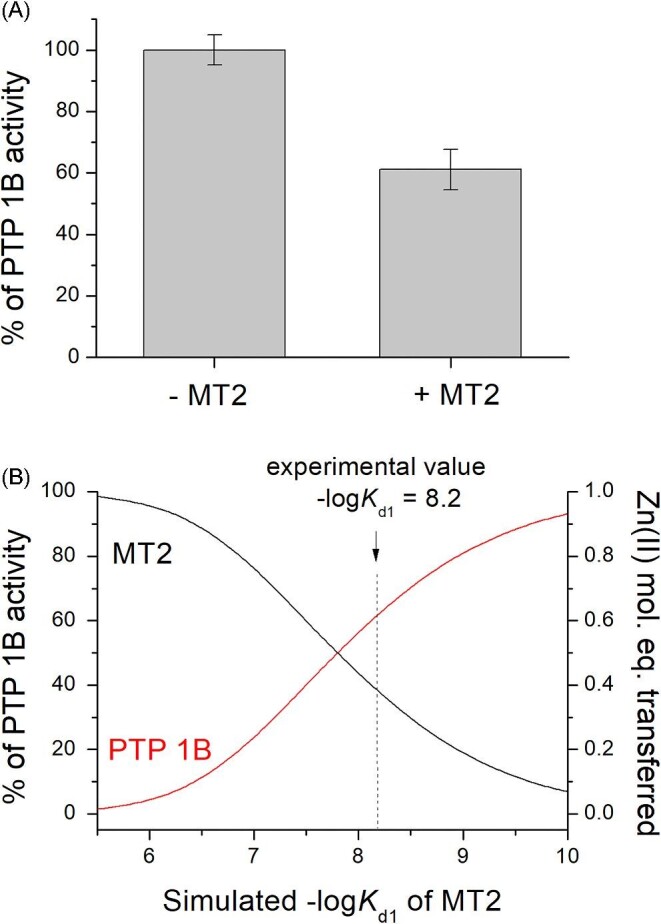
Inhibition of PTP1B by Zn(II) transferred from MT2. (A) Remaining activity of PTP1B (0.1 µM) incubated with 0.1 µM MT2 over the period of 60 min in 50 mM Na^+^-HEPES buffer, pH 7.4, 25°C assayed by *p*NPP; (B) Simulation of Zn(II) transfer to PTP1B from MT2 with various hypothetical –log*K*_d_ values of the weakest Zn(II)-binding site.

At the first step, PTP1B purchased from a commercial source was purified from DTT and EDTA, which was commonly added to maintain the enzyme in active form. After removing these agents (see Experimental procedures), 0.1 µM PTP1B was first examined regarding its activity with *p*NPP, then the same amount of PTP1B was mixed with 0.1 µM of freshly prepared MT2, and the sample was left for 1 h. After that period, it was assayed for its activity. Four independent measurements showed activity at 61% on average in comparison to the enzyme that was not incubated with MT2 (Fig. 7A). Similarly to the previous approach, calculation of Zn(II) transfer to PTP1B has been performed for a hypothetical zinc site of metallothionein with various affinities, –log*K*_d_ varying from 5.5 to 10.^93^ Figure 7B presents a comparison of such a simulation with the fraction of inhibited (and still active) PTP-1B under the applied conditions. It shows that the weakest Zn(II) site of MT2 must have –log*K*_d_ = 8.2 to be able to inhibit PTP1B to the experimentally observed level (Table 2). It should be noted that in this competition, only ∼0.6 Zn(II) mol. eq. was transferred to the enzyme. It means that the obtained *K*_d1_ value is probably not affected by Zn(II) transfer from other, more tightly bound sites in MT2. The obtained value fits the trend of previous approaches very well. Furthermore, previously performed studies have shown that apo-SDH (*K*_d_ = 6.3 × 10^−12^ M) is fully activated by even small fractions of MT, indicating the transfer from several zinc sites of MTs. It means that a tightly bound enzyme (or zinc structural site) with picomolar or even higher Zn(II) affinity is too strong to compete with the weakest site of MT2, causing its full transfer (Table 2).^48^ Such an enzyme should be suitable as a competitor for the tightest Zn(II)-binding sites of MT2.

### MT2 competition with weak low-molecular weight ligands

**Fig. 8:**
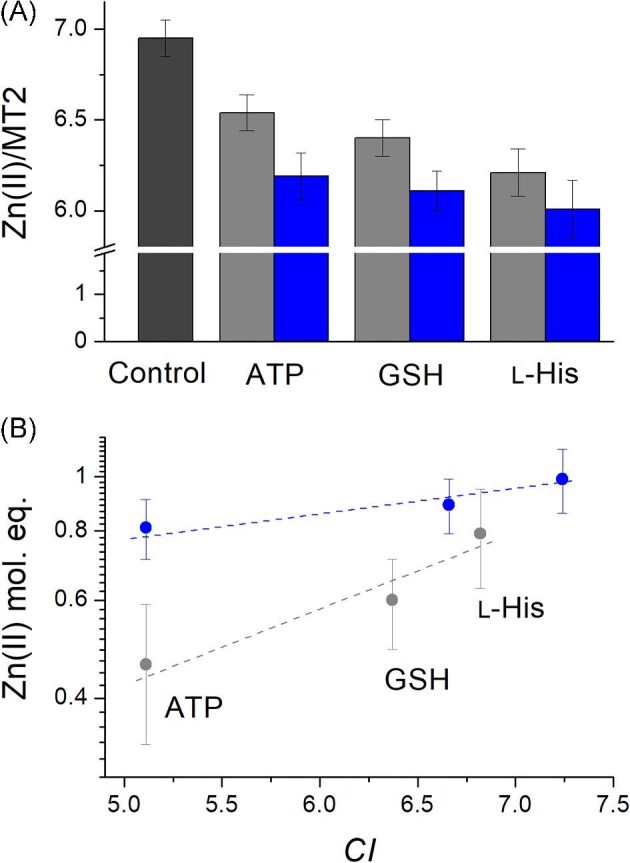
Analysis of Zn(II) transfer from MT2 to low-molecular weight cellular Zn(II) ligands. (A) MT2 (38 µM) was incubated with 0 (dark gray bar), 20 (gray bar), or 40 mM (blue bar) ATP, GSH, and L-His >60 min and separated by SEC in 20 mM Tris, pH 7.4, *I* = 0.1 M, 25°C. (B) Comparison between Zn(II) mol. eq. transferred and *CI* of low-molecular weight ligands. *CI* is the logarithm of the apparent dissociation constant of ZnZ complex [Zn(II) complex of theoretical molecule Z], where (ZnZ) is the sum of all Zn_i_H_j_L_k_ complexes of a particular competing ligand regardless of their stoichiometry and protonation states (see footnote of Table 3 for more details).^82^

In order to demonstrate the importance of the weak Zn(II)-binding site in metallothionein in the context of low-molecular weight biomolecules, we performed a competition study using ATP, GSH, and L-histidine (L-His). All these molecules have been shown to bind Zn(II) with relatively weak or medium-weak affinities.^82^ Although these ligands do not bind Zn(II) tightly, they are present in the cell at relatively high (L-His) or very high concentrations (GSH, ATP). Therefore, their participation in the cellular zinc pool is a subject of scientific discussion.^98^ So far, in this report, we have presented how moderate (nanomolar) or high (picomolar or femtomolar) affinity ligands interact with MT2. Now, we test whether low-molecular-weight (LMW) ligands may affect loosely bound Zn(II) of MT2 and how efficient such competition might be. For that purpose, 38 µM MT2 samples were incubated with 20 or 40 mM ligand for 1 h. Then samples were subjected to analytical SEC to separate Zn(II)-depleted MT2 and LMW fractions with bound Zn(II). To determine how much Zn(II) is present in the protein fraction, DTNB and PAR spectroscopic assays were performed (see Experimental procedures). Figure 8A shows the metal content of MT2 fractions in proteins separated after incubation with a ligand compared to the control sample, which was not incubated with any ligand. The most efficient Zn(II) transfer of 0.99 Zn(II) mol. eq. was noted for 40 mM L-His, while the lowest, ∼0.46 Zn(II) mol. eq., was noted for 20 mM ATP, which is expected based on the high- and low-Zn(II) affinities of these ligands, respectively. Unfortunately, due to the various stoichiometry of Zn(II) complexes formed by L-His or GSH, direct comparison of transferred Zn(II) with their dissociation constant is impossible. To overcome this problem, the simplification of the system stoichiometry can be made by introducing the competitivity index (*CI*), which, due to its definition, varies slightly for different concentrations of ligands (see Fig. 8 for more details).^81,82^ Figure 8B indicates a tendency between Zn(II) mol. eq. transferred from MT2 and *CI* ligand values. The linearity indicates that the transfer observed in this experiment occurs from a single, weak Zn(II)-binding site. Mobilization of the second and next Zn(II) would require stronger competitors. However, an important issue is that physiological LMW ligands may possibly participate in Zn(II) transfer from mammalian MTs under certain conditions (see discussion below).

Since LMW zinc ligands outcompete MT2, there is a question of whether these reactions may be applied for the determination of weakly bound Zn(II) in MT2 as well. It can be achieved using the approach for PAR competition, but with slight modifications due to Zn(II)–LMW complex stoichiometry. The calculation requires the introduction of a hypothetical Z ligand with a 1:1 Zn(II) complex stoichiometry. Therefore, *K*_d1_ of Zn_7_MT2 is now defined by Equation (13), where *K*_d_^ZnZ^ is an apparent dissociation constant of the ZnZ complex under conditions strictly used here, which is calculated as 10^−^*^CI^* since CI according to its definition is –log*K*_d_^ZnZ^.


(13) \begin{equation*}{K}_{{\mathrm{d}}1} = {K}_{\mathrm{d}}^{{\mathrm{ZnZ}}} \cdot \frac{{\left[ {{\mathrm{Z}}{{\mathrm{n}}}_6{\mathrm{MT}}2} \right] \cdot \left[ {{\mathrm{ZnZ}}} \right]}}{{\left[ {{\mathrm{Z}}{{\mathrm{n}}}_7{\mathrm{MT}}2} \right] \cdot \left[ {\mathrm{Z}} \right]}}.\end{equation*}

Based on the transferred Zn(II), all reactant concentrations are calculated, and the obtained –log*K*_d1_ value is 7.9 ± 0.2, 8.9 ± 0.1, and 8.7 ± 0.3 for ATP, GSH, and L-His, respectively. An average –log*K*_d1_ value from the entire experiment is 8.5 ± 0.5, which is again convergent with all approaches used so far (Table 2). Considering the results of all the solution-based experiments described above, we have calculated an average value of the dissociation constant (–log*K*_d1_) for the weakly bound Zn(II) in human MT2 to be 8.4 (Table 2).

### Monitoring of MT2 competition with ZFs by ESI-MS

Electronic spectroscopies were the most frequently used techniques for the characterization of metal-binding properties, structure, and thermodynamics of metallothioneins. However, it should be noted that many observable effects reflect an average picture of several states and processes occurring in the solution. An alternative method of higher resolution that allows in-depth analysis of variously metallated species is MS, which is an excellent method for structural and even thermodynamic characterization of multi-species systems such as MTs.^24–26,28–30,33,50,99–101^ It must be taken into account that the picture observed in the ESI-MS spectrum, the most frequent ionization MS technique, is a snapshot captured in the gas phase, which does not necessarily quantitatively represent the speciation in water media.^30,102–104^ However, several ESI-MS-based reports on MTs have shown that relative intensities of MT species or their competitors were quantitatively converted to the speciation in the solution and even applied for determining MT1 Zn(II) complexes’ dissociation constants.^50,105^ These values, despite minor differentiation, show picomolar Zn(II) affinity for all seven zinc sites, which is in contrast to the solution studies presented so far in this report. Therefore, here, we focused on determining whether this technique can be successfully used to study the affinity of the weak-binding site of Zn(II)-MT2 in the solution and how MS-obtained values correspond to spectroscopic ones.

**Fig. 9:**
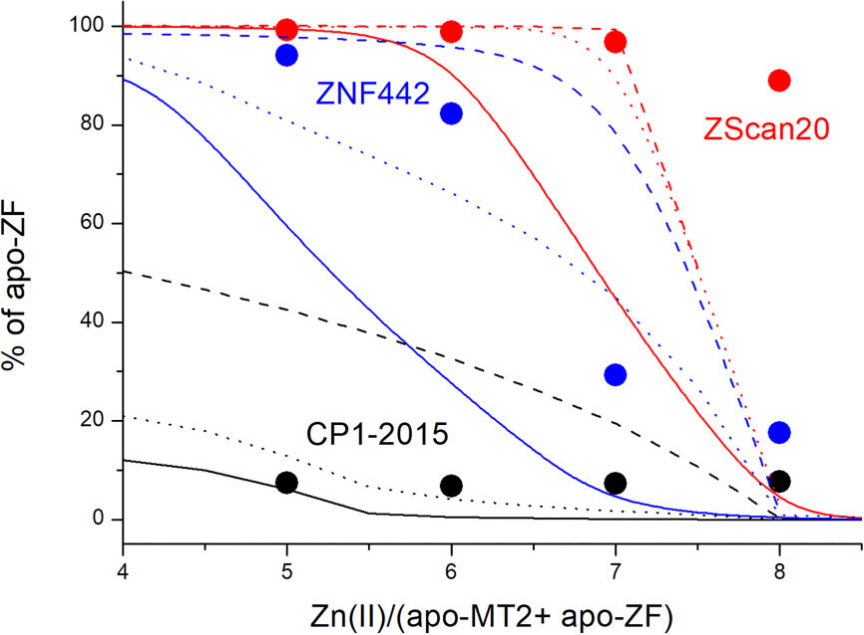
Competition of ZF domains of various affinities with apo-MT2 (T) for Zn(II) performed using ESI-MS. ZScan20, ZNF442, and CP1-2015 ZF peptides (30 µM) were mixed with 30 µM apo-MT2 in 100 mM ammonium acetate (pH ∼ 7.5) followed by addition of ZnSO_4_ to reach a metal-to-(apo-T + apo-ZF) molar ratio of 5–8. Samples were incubated for 1 h under anaerobic conditions, and then spectra were recorded. Circles demonstrate fractions of apo-ZF at particular Zn(II)/(apo-T + apo-ZF) molar ratios. Solid, dashed, and dotted lines indicate fractions calculated based on known ZFs and MTs affinities to Zn(II).^30,32,50,53,60,76^

In this approach, we used three different ZF domains, CP1-2015 (CCHH), ZNF442, and ZScan20, representing gradually decreasing zinc affinities. –log*K*_d_ values of their Zn(II) complexes are 12.3, 10.4, and 7.9, respectively.^60,76^ Choosing these ZF domains, we also wanted to test how the determination of MT2’s Zn(II)-binding affinities is related to the competitor efficiency for Zn(II) chelation. In all cases, an equimolar (30 µM) mixture of metal-free ZF and MT2 (thionein) in 100 mM ammonium acetate was titrated with ZnSO_4_ starting from 5 to 8 Zn(II) mol. eq. over the mixture of metal-free ZF and MT2. It resulted in the formation of a series of variously metallated MT2 species. However, in quantitative peak height analysis, ZF saturation was taken into account for simplicity, similarly to previous studies (Fig. S3).^50^ For example, when 6 Zn(II) mol. eq. were applied, 100% of metal-free ZScan20, 73% of ZF422, and 9% of CP1-2015 were detected (Fig. 9). For 7 Zn(II) mol. eq., the percentages of metal-free ZFs were 95, 30, and 8%, respectively. This trend was expected considering ZnZF complexes’ stability order; however, saturation of ZNF442, especially ZScan20, was significantly lower than the value resulting from the dual nanomolar and picomolar affinity model of MT2 (solid lines).^32^ The saturation profile of those ZFs much more closely matches the Zn(II)-MT1 model taken from previous ESI-MS investigation, where only picomolar affinity sites were found (dashed lines).^50^ Better overlapping is observed even for the human MT2 stability model (dotted lines) adopted from our recent ESI-MS studies, for which affinities of all Zn(II) ions also vary in the picomolar range.^30^ Table 2 presents the best –log*K*_d_ values of the weakest zinc site of MT2 estimated here from the ESI-MS competition for ZScan20 and ZNF442 ZFs. The stability of the Zn(II)-CP1-2015 complex is too high compared to the weak affinity site of MT2, and therefore competition was not observable [CP1-2015 ZF was saturated by Zn(II) in almost all experimental conditions]. In other words, data obtained from the ESI-MS competition experiment indicate that the weakest Zn(II)-binding site in MT2 is much tighter than that in the whole range of experiments performed here and much better fits the ‘old’ model of all seven Zn(II) ions bound in MTs with the same affinity. As stated above, the difference in the affinities most likely stems from measurement in the gas phase rather than the solution-based approaches we utilized above.

### Weak and moderate zinc-bindings sites in MTs are obscured by high-affinity sites in MTs

As pointed out above, we performed the described experiments to (i) indicate difficulties in the determination of nanomolar and picomolar affinities of mammalian MTs, (ii) present and discuss advantages and disadvantages of various approaches that might be useful for the determination of the various affinity binding sites, and (iii) show how the presence of tight binding site(s) may obscure experimental signals from weaker site(s) to such an extent that it interferes with observing the presence of weaker sites.

Historically, the method of choice used for the characterization of metal-to-metallothionein binding was, and still is in the case of newly investigated (from various organisms) MTs, UV-vis absorption spectroscopy. Titration of thionein with Zn(II) at neutral pH results in an almost linear absorption increase in the UV range (maximum ∼218 nm). This increase is linear up to ∼4 Zn(II) mol. eq., while at additional Zn(II) mol. eq., it becomes slightly rounded.^32^ Extrapolation of the linear trend and maximal absorbance gives a Zn(II)/MT2 ratio that is <7 Zn(II) mol. eq., in contrast to Cd(II).^30^ A much clearer difference between the coordination of those metal ions is observed for pH titration of MTs. Cd(II) metalation occurs in two steps, in which first the α-domain and then the β-domain are filled, but the process is not fully cooperative.^18,30,43^ The corresponding Cd_x_S_y_ clusters are formed with significant cooperativity, which is observed in sharp metal-to-thionein titration but also pH titration curves. However, our recently published MD simulations and MS-based proteomics analysis indicated that this process is not fully cooperative.^30^ For Zn(II), the huge absorption increase at the beginning of metalation corresponds to the binding of 4 Zn(II) ions into α- and β-domains (two ions per domain with low-picomolar affinity) to ∼14 Cys residues without extensive clustering. The rest of the Zn(II) ions fill both domains (preferentially α-one) with high-picomolar and nanomolar affinities in such a way that both clusters are filled, which is noticeable in the modest hypsochromic shift of LMCT bands.^32^ The formation of both clusters in the second metalation step turns out to also distinctly lower the absorption increase, and it is the explanation why the extrapolation end point is not present at 7 Zn(II) mol. eq. to apo-MT2 molar ratio. This stepwise cluster formation, in contrast to the more cooperative manner for Cd_x_S_y_, is one reason for the weaker binding of subsequent Zn(II) ions to MTs.^30,106^ The last, weakest Zn(II) ion was found to bind to the β-domain, and deprotonation of the Cys21 residue accompanies this process. Taking into account the whole metalation process described above, in pH Zn_7_MTs titrations, the most dominant is an absorption increase from tightly bound Zn(II) ions, while moderate and loosely bound ions are observable as a smooth absorption increase over a wider range of pH, causing the whole titration curve to be asymmetrical (see Fig. 18 in ref [2]). Therefore, treating this pH-dependent process in which all Zn(II) ions are bound with the same affinity and taking this curve symmetrically was a source of miscalculation of stability constants. The assumption that all cysteinyl thiols have the same p*K*_a_ values is an additional factor that complicates the determination of Zn(II) binding/dissociation constants.^38,44^ These results more likely relate to the affinities of the tightest zinc sites and do not represent actual affinities of moderate or weak zinc sites. Their appearance is much less appreciable in pH-dependent UV-vis titrations due to lower LMCT molar absorption coefficients accompanying formation of such sites.^30^ Overall, although UV-range absorption electronic spectroscopy is highly helpful for the characterization of Zn(II) (un)binding to thionein, the obtained results should be treated with caution since particular Zn(II) ions bind with different affinities and spectral properties. Simplification of the system leads to a misunderstanding of protein functions (see below).^2^

## Guideline for future experiments on MT and multiple metal-binding proteins

**Fig. 10:**
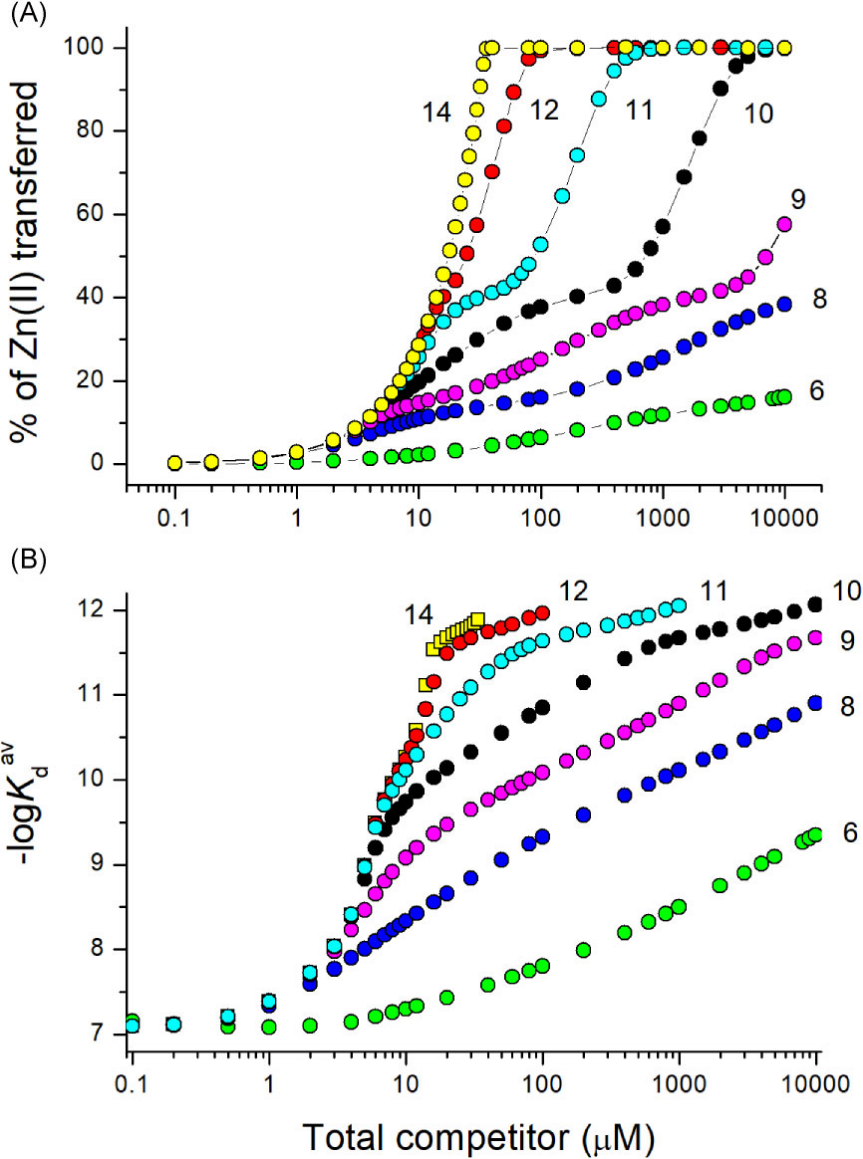
Simulations of Zn_7_MT2 competition with hypothetical chelators with gradually changed *K*_d_^probe^ from 10^−14^ to 10^−6^ M. (A) Relationship between competitor total concentration and percentage of Zn(II) transferred from MT2. (B) Average dissociation constants (*K*_d_^av^) that are calculated at various competitor concentrations. Calculations were performed for the commonly used simplified stability model, according to which all Zn(II) ions are bound with independent and undistinguished affinities.

Spectroscopic competition experiments were among the most widely applied approaches over the years for the determination of Zn(II) affinity in MTs. Regardless of the stoichiometry of competitors, they report the amount of Zn(II) transferred under steady-state conditions. In a typical experiment, excess of a zinc competitor (a probe or non-chromogenic chelator) is mixed with fully loaded Zn(II)-MT and left for equilibration. The signal response is then converted to Zn(II) competitor complex concentration using either the molar absorption coefficient, calibration, or atomic detection. Finally, the most important issue requires choosing the affinity model of Zn(II)-MT. The simplest and the most often chosen model is that where all seven Zn(II) ions are bound with the same undistinguished high affinity. It allows *K*_d_^av^ calculation based on a single Zn(II) transfer experiment (e.g. Equations 3–5). It should be, however, noted that the percentage of Zn(II) transfer depends on competitor affinity for Zn(II) and its total concentration (Fig. 10A). It is an important issue since it affects the final *K*_d_^av^ value determination due to the chosen simplified model. In fact, Zn(II) ions are not bound with the same affinity, and the number of mobilized Zn(II) ions and their affinity for MT are reflected in the calculated *K*_d_^av^ value, as presented in Fig. 10B. For instance, the use of weakly bound competitors, such as ATP, L-His, and GSH applied in this study, mostly results in mobilization of the weakest zinc site. Therefore, using a simplified model, one will calculate a *K*_d_^av^ value in the low-nanomolar range. The use of a competitor with *K*_d_ ∼10^−8^ M or slightly tighter, depending on the used concentrations, allows 1 to ∼3 Zn(II) ions to be removed from MTs. Then, *K*_d_^av^, calculated as mentioned above, represents, in a sense, an average value of dissociation constants of mobilized Zn(II) ions, as presented in Table 3.

**Table 3.** Average dissociation constants of MT2 that can be determined in the competition studies after mobilization of 1–7 Zn(II) ions. Values and stability model (*K*_d1–7_) were taken from ref. [32]

Mobilized zinc sites	Percentage of mobilized Zn(II)	*K*_d1–7_ of mobilized sites	Determined *K*_d_^av^
Weak site	14.3	*K*_d1_	10^−8^ M
Weak site + one moderate site	28.6	*K*_d1_ + *K*_d2_	1 × 10^−10^ M
Weak site + two moderate sites	42.9	*K*_d1 +_ *K*_d2 _+* K*_d3_	3 × 10^−11^ M
Weak + two moderate + four tight sites	100	*K*_d1 +_ *K*_d2 _+* K*_d3 _+ 4*K*_d4-7_	3 × 10^−12^ M

**Fig. 11:**
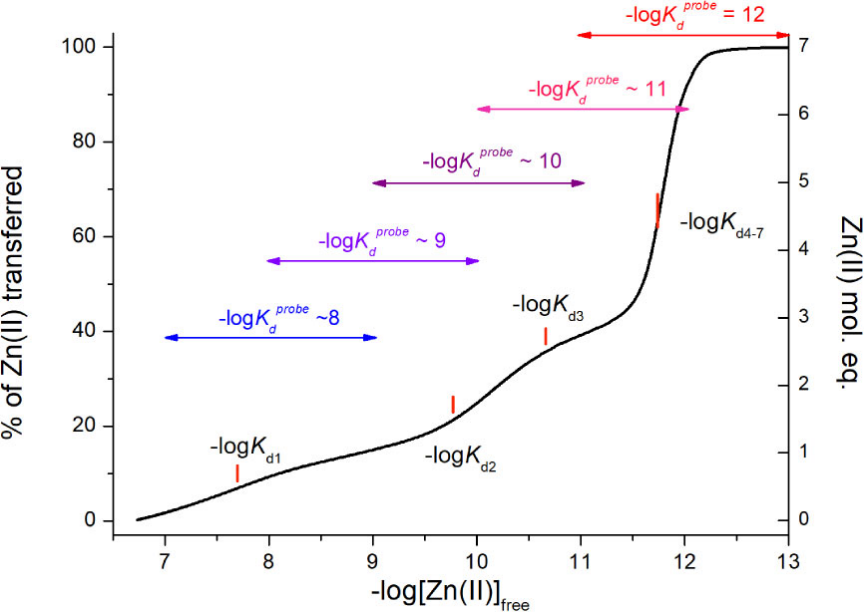
Simulations of Zn_7_MT2 competition with hypothetical chelators with gradually changed *K*_d_^probe^ from 10^−14^ to 10^−6^ M. The graph describes the relationship between competitor total concentration and percentage of Zn(II) transferred from MT2. Calculations were performed for Zn(II)-MT2 stability model from ref.^32^

The application of a very tight competitor (for instance, DTPA, diethylenetriaminepentaacetic acid) makes it easy to remove all seven Zn(II) at a relatively low concentration. Therefore, a competition experiment would result in obtaining/calculating a *K*_d_^av^ value close to the most tight Zn(II) ions in MT (Table 3). A competitor with slightly weaker affinity does not remove all Zn(II) ions, which results in a higher *K*_d_^av^ value obtained in such an experiment. To further visualize this effect, we performed simulations presented in Fig. 10 and Table 3 to explain why experimental *K*_d_^av^ obtained in a series of experiments over the years varied significantly. Its values, according to the adopted assumption, are closely related to the probe and its concentration. Therefore, the most appropriate approach is to consider the fact that all Zn(II) ions in the protein bind with different affinities. Then taking several competitors (zinc chromogenic probes or non-chromogenic chelators) with gradually increasing Zn(II) affinities would be the most optimal to determine affinities of all zinc sites in a wide –log[Zn(II)]_free_ range without obscuring low-affinity sites. Figure 11 presents the relation between the percentage of Zn(II) transferred to competitors and free Zn(II) concentration in a wide 10^−7^–10^−13^ M range. It should be underlined that the obtained isotherm is competitor-independent, meaning that this relationship is the same regardless of affinity or Zn(II) complex stoichiometry of applied chelators. Obviously, such an experiment is not trivial and requires several competitors or probes with *K*_d_^probe^ varying from 10^−7^ to 10^−13^ M, taking into account their standard sensitivity {–log[Zn(II)]_free_ ± 1 of –log*K*_d_^probe^}. However, increasing this sensitivity to higher than ±1 of –log*K*_d_^probe^ allows one to use lower numbers of probes or even one ultrasensitive probe, as in the case of FluoZin-3, where *K*_d_^FloZin-3^ is 8.9 nM.^32,107,108^ It allows one to measure free Zn(II) concentration in the range from micromolar to subpicomolar (see Equation 12), when it is used in micromolar concentrations. Notably, ZnAF-2F has similar properties and can be used instead of FluoZin-3.^62^ Results on the weak binding site of Zn_7_MT2 presented in this article are convergent with data obtained for FluoZin-3 competition for the same protein.^32^ It should be added that in the determination of Zn(II) affinity of a particular MT, usage of metal-free thionein should also be considered. Its titration with Zn(II) in the presence of a zinc probe (especially one that is ultrasensitive) allows the necessary range of free Zn(II) concentrations to be covered in one experiment, which is impossible when only fully Zn(II)-loaded MT is applied.^32^ Obtaining the free Zn(II) concentration relation versus transferred Zn(II) or its equivalents (Fig. 11) allows one to get deeper insight into the stability model of particular MTs. For MT2, there are indeed four zinc sites with low-picomolar affinities. Characterization of the remaining three sites requires special attention; however, there is a clear difference between one weak and two moderate affinity sites (Fig. 11).

Conclusions coming from spectroscopic competition of metal chelators and Zn(II)-MTs are also accurate and worth using in the case of application of other methods or techniques where their equilibration with MTs is practiced. For instance, *ITC* has been used for the determination of Zn(II) ions’ affinity for mouse MT3. In this approach, assuming independent and metal binding with the undistinguished affinity *K*_d_^av^ value of 2.5 × 10^−12^ M was obtained.^51^ It should be noted that this value was determined by Zn_7_MT3 competition with the high-affinity chelator DTPA (*K*_d_^DTPA ^= 5.0 × 10^−15^ M, pH 7.4).^109^ The same Zn_7_MT3 investigated with EDTA at lower pH 6.0 (*K*_d_^EDTA ^= 1.0 × 10^−12^ M) indicated the presence of weak, moderate, and tight binding sites.^49^ Decreasing affinity of the competitor to the subnanomolar range more likely would indicate differentiation of zinc sites at neutral pH, which has been observed for MT3 using *chemometric-assisted voltammetric analysis*.^110^ In another approach, *^19^F-NMR spectroscopy* was applied for monitoring competition of 5F-BAPTA with Zn(II)-MT2 (3.2 × 10^−12^, pH 8.0) and MT3 (1.6 × 10^−11 ^M, pH 8.0). The obtained weaker average affinity of Zn(II) for MT3 than in the case of ITC analysis (taking account of the pH difference) indicates variations between used techniques. The difference may also derive from the fact that 5F-BAPTA binds Zn(II) with much weaker affinity (*K*_d_^5F-BAPTA ^= 2.3 × 10^−10^ M, pH 8.0) and does not remove all Zn(II) ions during competition, which leads to a higher *K*_d_^av^ value of zinc sites in MT according to the above discussion (Table 3, Fig. 10). This is further proof that the assumption of undistinguished affinities of all Zn(II) ions in MTs is not proper and leads to either overestimation or underestimation of stability constants and related misunderstandings, forming the subject of many scientific discussions. Therefore, we strongly recommend not to use the simplified model but to study the whole process of Zn(II) (un)metalation in detail.

Another method that has been applied for metal affinity in MTs’ investigation is *MS* (especially *ESI-MS*), which very elegantly demonstrates the speciation occurring in the MT system during the formation of clusters or their reaction with other metal-binding molecules such as competitors. It should be noted, however, that this technique is qualitative rather than quantitative since ESI-MS-monitored speciation corresponds to protein ions speciation in the gas, not water, phase.^33^ Several physical processes such as supermetalization, pH drop, Zn(II) carry-over, various hydration of protein molecules in both phases, and various ionization of metal-free and bound protein affect the ESI-MS results compared to the solution state.^30,102–104,111,112^ Despite this, Pinter *et al.* used metal-free carbonic anhydrase, which binds Zn(II) tightly (–log*K*_d_^CA^ = 11.4), as a competitor of MT1.^50,105^ Data obtained at various Zn(II) to apo-MT1 + apo-CA ratios allowed for the determination of apparent dissociation constants of all zinc sites (Table 1), similar to each other (–log*K*_d_s from 11.8 to 12.5), suggesting that MT1 binds Zn(II) tightly. Also, our recent study on Zn(II) binding to MT2 performed by the same method has shown a similar (slightly weaker) zinc site affinity pattern (–log*K*_d_s from 10.6 to 12.0).^30^ Similarly, data on ZFs’ competition with MT2 performed here using peptide competitors indicate that the *K*_d_^av^ of the weakest MT2 site is in the low-picomolar range (Table 2). Overall, all data show that affinities of Zn(II)-MTs determined by ESI-MS are significantly elevated compared to data from spectroscopic approaches obtained in this and previous studies. This issue is the topic of several recent studies, where the authors indicate that speciation in the water and gas phases does not correlate with each other due to physical differences between the species. Regarding MTs, especially useful in the discussion on this topic were MD calculations of variously Zn(II)-loaded MT species supported by ion-mobility MS measurements. They show that the significant difference in molecule hydration in both phases and differences in the intramolecular (including interdomain) hydrogen network are the major reasons for the elevation of zinc sites determined in the gas phase by MS.^30,33^ Therefore, all stability data obtained via this method should be treated with caution and may lead to inappropriate conclusions about the metal-binding properties of metallothioneins.

### Factors influencing MTs’ composition, stability, and MT-involved investigations

Searching for metallothioneins’ functions took more than half a century and is still under investigation. There are many reasons for that, but the most important one is the lack of rigid structure, which led to numerous problems in biochemical investigations. Lack of rigid structure causes the increase in protein reactivity. High flexibility decreases metal selectivity, which is in opposition to structural and enzymatic metal sites. This and the cellular presence of more than one isoform (MT2 and MT1 subisoforms coexist in all tissues) make MTs highly heterogenous proteins, which are difficult to study not only in tissues but even in purified forms. It should be underlined that all chemically different MT forms, for example isoforms, modified or oxidized proteins, etc., demonstrate different properties in metal binding, formation of particular sites, and thermodynamic stability. Therefore, it is critical for any kind of characterization to use a protein with defined and pure chemical form. Only then can the obtained parameter values reflect the actual properties of the defined MT form.

Most problems in the characterization of physicochemical/biophysical properties of MTs stem from simplified or not sufficiently sensitive approaches, which have been demonstrated and discussed in the previous sections. However, the way that MTs are produced is another reason for obtaining non-convergent or even opposite data. The most common and elegant way for production of the purest (sequentially) form is recombinant MT production in a bacterial system. It avoids most of the posttranslational modifications that could be added in other protein production systems.^2,64,65^ However, some reports show that even in bacteria, MTs may be at least partially posttranslationally modified under certain conditions.^63^ Therefore the use of established production systems is highly recommended. Due to the fact that MTs are metalloproteins and are purified in several steps, it is of high importance to take care of all stages of protein production and its reconstitution. In some cases, MTs are produced in bacteria as Cd(II)-loaded proteins when Cd(II) salts are added to bacteria's growth culture. Their chemical transformation to the Zn(II)-loaded state requires thionein production in acidified conditions, similarly to our approach, and subsequent Zn(II) reconstitution.^45^ Besides the fact that Cd(II) may increase the production yield in bacteria, it protects MTs against oxidation since Cd(II) is bound tighter than Zn(II) if that is added during bacterial growth.^2^ Acidification of bacteria-produced MTs was indicated as a factor that may affect MT properties.^40^ In this study, we observed that saturation of metal-free human MT2 at slightly basic pH (pH 8.0) compared to a standard protocol covering thionein purification in acidic conditions and further metal loading results in the same protein redox state and physicochemical properties. However, MT acidification to very low pH, for example, <0, affects MT metal-binding properties. Nevertheless, such an extreme pH would change the properties of almost any protein. The pH of Zn(II)-loaded protein purification [from Zn(II) excess, for instance] is also an important issue because of nanomolar binding properties of the weakest site. SEC filtration at neutral pH increases the chance of partial loss of the weakest site during protein production. Slightly increasing pH to 8 or even 8.6 as in this study causes that Zn(II) binds protein more tightly and is still bound to the protein.^2^ The addition of the reducing agent is not necessary when Zn(II) or Cd(II)-loading protein is prepared from an acidified thionein and metal salt mixture by gentle pH increase under a neutral gas blanket.^57^ However, addition of TCEP, very weakly Zn(II)- and Cd(II)-binding disulfide reducing agent, does not affect the metal-load state (in the case of zinc and cadmium proteins) and protects proteins during purifications. However, the presence of TCEP in running buffer may influence subsequent readouts of total thiols since TCEP reacts with DTNB or DTDP.^32,61,113^ All of the factors indicated here or preparation steps may influence the homogeneity and quality of the final protein. MT production in any research requires a metal-load state analysis. Usually, the concentrations of metals, thiolates or sulfur coming from ICP studies are provided in research reports. In Zn(II)-MTs, the molar ratio between Zn and S content measured by ICP is used as proof of protein quality. However, it should be underlined that this factor alone may not represent protein quality since similar element ratios may be found for partially metal-loaded and partially oxidized/modified proteins. It is especially important for MT samples produced from animal tissues to additionally use spectroscopic assays besides ICP analysis. In that case, control of all homogeneity factors is either very hard or impossible to accomplish.

### Presence of weak and moderate binding sites in mammalian MTs determines their zinc buffering function

Affinity for metal ions was studied from the very beginning of MT discovery.^2^ As mentioned above, it was believed for many years that all seven Zn(II) ions are bound to MTs with the same, non-distinguished high affinity. This model considered only coexistence of saturated Zn_7_MT and thionein species, which occur only at low or even subpicomolar levels. The transformation of both these species occurs in a very narrow and low range of free Zn(II), unlikely for cellular events. Several findings concerning MTs’ quantification in tissues and cell lines have indicated that cells contain a surplus of tight and moderate Zn(II)-binding ligands.^31,114^ Moreover, isolated MT fractions were capable of external Zn(II) binding, indicating their not fully saturated status. Considering the fact that only Zn_7_MT and apo-MT (T) coexist, it would mean that significant amounts of thionein must be present in isolated protein samples. For instance, the T percentage in rat liver, kidney, brain, and testis was found to be 27, 54, 53, and 9% over total protein (Zn_7_MT + T), respectively.^115^ Similar results were found in HT-29 colorectal cancer cells, where T presence was found to be 30% and its level decreased when cells were incubated with increasing concentrations of ZnSO_4_.^31^ Enormous progress in the development and applications of various types of fluorescent zinc probes and sensors has shown that free Zn(II) concentration is far from the expected low-picomolar range and varies from subnanomolar to nanomolar range (10^−10^–10^−9 ^M). However, lower and higher concentrations have also been found.^2,116^ A very important step in understanding MTs’ nature was made when an ultrasensitive fluorescent FluoZin-3 probe was applied to human Zn(II)-MT2.^32^ As extensively discussed above, it showed the presence of weak, moderate, and high-affinity zinc sites in one protein. This feature makes this protein unique since it is able to bind (accept) and serve (donate) Zn(II) ions with various affinities, from picomolar to nanomolar.^1^ As a consequence, depending on cellular free Zn(II) concentrations, various Zn_4-7_MT species may be present due to the wide range of MTs buffering properties (in contrast to original model). It indicates that fully loaded Zn_7_MT is not the only species, as postulated before, and the presence of moderate and weak binding sites extends the zinc buffering range. It shows that the divergent Zn(II) affinities of MTs determine their important function in Zn(II) handling and buffering. The concept of Zn_4–7_MT species being major cellular zinc buffering components in a wide range of available free Zn(II) is still under investigation by our and other groups. Recent structural studies have shown that the most important factors that differentiate the affinity of zinc sites is the mechanism of their formation (tight sites differ from moderate and weak ones), the hydration of particular Zn_x_MT species, and the intramolecular hydrogen bond network, including interactions between domains.^28–30,33^

Saying about MTs’ functions, one must remember their structures. It should be emphasized that the common Zn_7_MT form is not the only one formed in cellular conditions. Under normal once, as discussed above, Zn_4–6_MTs species are rather formed and are responsible for Zn(II) buffering. The structures of Zn_4–6_MTs species differ from fully loaded ones and are very difficult to study due to their high conformational dynamics.^30,33^ So far, only MS has been highly informative regarding these dynamic species structures. Worth mentioning is that these partially saturated species are functional forms that interact with proteins and ligands during MT-protein or MT-ligand complex formation or Zn(II) transfer. Their structure and dynamics might be key factors during these processes. During Zn(II) transfer, another scenario also occurs, where Zn(II) ions dissociate from MT, forming less saturated species (the essence of buffering), namely making Zn(II) ions available that then bind to the apoproteins. Last but not least way of Zn(II) transfer is MTs interaction with the LMW ligand pool, in which components (e.g. GSH) concentrations may vary, modulating Zn(II) load in MTs and metalloproteins.

As we have shown, the buffering concept of MTs and experimental approaches for the investigation of proteins are related to each other and have evolved over the years. The first proposed MT function is different from the current one and was obviously limited to what could be concluded from the first experimental approaches. The concept of only Zn_7_MT and T species being present in the cell hinged on the assumption of the same affinity for all zinc sites. It simply derived from methodological limitations in the investigation of all seven sites of MTs. Progress in the field of zinc probes allowed light to be shed on zinc biology and our understanding of what free Zn(II) is and what information its value and change hold.^117,118^ It shows that our biochemical perception of metallothionein functions was and still is strictly related to the experimental possibilities, including high-resolution approaches and understanding of metals’ equilibria and their importance.

## Conclusions

The data presented here demonstrate that the determination of weak and moderate Zn(II)-binding sites in metallothioneins and related proteins must be performed with caution. The presence of four high-affinity sites and the way of binding to MTs strongly determine the picture observable in spectroscopic studies. Similarly, four of the seven zinc sites in the competition studies often obscure the presence of weak sites due to the fact that average, not step-binding constants are determined. The current data and conclusions, convergent with our previous studies performed with the FluoZin-3 probe, show that the simplification of data processing due to the assumption that all Zn(II) ions must bind with the same affinity proved to be the most important problem in correct determination of metal-protein equilibria. It also strongly affected the understanding of MT functions for many years. We also show that superficial use of MS can provide inaccurate results on metal-binding affinity. This article demonstrates how the limitations of applied methodology over the decades have affected the perception of MTs function, which has changed from tight metal binding only (storage function) to one that is highly dynamic (regulatory function).^32^

## Supplementary Material

mfad027_Supplemental_File

## Data Availability

The data underlying this article are available in the article and in its online supplementary material.
