# Supplementary material for: From methodological limitations to the function of metallothioneins - a guide to approaches for determining weak, moderate, and tight affinity zinc sites
Source: Metallomics. 2023 Apr 27;15(5):mfad027. doi: 10.1093/mtomcs/mfad027 (PMC10210066; doi:10.1093/mtomcs/mfad027)
Supplement: mfad027_Supplemental_File [file mfad027_supplemental_file.docx]

*Supplementary information*

From methodological limitations to the function of metallothioneins - A guide to approaches for determining weak, moderate and tight affinity zinc sites

Adam Pomorski,^1^ Agnieszka Drozd,^1^ Anna Kocyła,^1^ Artur Krężel^1^*

*^1^Department of Chemical Biology, Faculty of Biotechnology, University of Wrocław, Joliot-Curie 14a, 50-383 Wrocław, Poland*

Correspondence to: Artur Krężel, e-mail: artur.krezel@uwr.edu.pl

**Materials.** ZnSO_4_·7H_2_O, NaClO_4_·H_2_O (4-(2-hydroxyethyl)-1-piperazineethanesulfonic acid (HEPES), Tris base, tris(2-carboxyethyl)phosphine hydrochloride (TCEP), L-histidine, adenosine triphosphate (ATP), reduced glutathione (GSH), ethylenediaminetetraacetic acid (EDTA), HCl (trace metal grade), TFA, Zincon monosodium salt, ZnAF-2F (zinc fluorescent probe), sorbitol dehydrogenase (SDH) from ship liver were purchased from Sigma-Aldrich (Merck). Trypton, yeast extract, LB Broth, agar, agarose, isopropyl-β-D-1 thiogalactopyranoside (IPTG), SDS were from Lab Empire, NaCl, KNO_3_, NaOH, glycerol, KH_2_PO_4_·H_2_O_,_ K_2_HPO_4_ from POCH (Gliwice Poland), pTYB21 vector and chitin resin from New England BioLabs, human recombinant protein tyrosine phosphatase from Biaffin GmbH & Co KG, 5,5'-dithiobis-(2-nitrobenzoic acid) (DTNB) from TCI Europe N.V., DL-dithiothreitol (DTT) from Iris Biotech, Chelex 100 resin from BioRad. Fmoc-protected amino acids, piperidine and HBTU were purchased from Iris Biotech GmbH. All of the experiments were performed in chelexed buffers and solutions. All buffers were prepared with milli-Q water obtained with deionizing water system (Merck Millipore, USA).

**Table S1.** Amino acid sequences of peptides and proteins used in this study.

| Peptides or protein | Amino acid sequence |
| --- | --- |
| ZScan20 | Ac-KPYKCLGSGKSFSDRANLSTHQRIHIGEK-NH_2_ |
| ZF422 | Ac-KRYDCKECGKTFSSSGNLRRHIIVQRGGG-NH_2_ |
| CP1 | Ac-YKCPECGKSFSQKSDLVKHQRTHTG-NH_2_ |
| ZF133-11 | Ac-PMVCGECGRGFSQKSNLVAHQRTHSGER-NH_2_ |
| ZF133-11 C7E | Ac-PMVCGE**E**GRGFSQKSNLVAHQRTHSGER-NH_2_ |
| human MT2 | MDPNCSCAAGDSCTCAGSCKCKECKCTSCKKSCCSCC  PVGCAKCAQGCICKGASDKCSCCA |

**Table S2**. Experimental and calculated molecular masses of peptides and recombinant protein obtained in this study and determined by mass spectrometry. Ac- and -NH_2_ denote acetyl and amide functions added during peptides synthesis.

| Peptides or protein | Experimental MW | Calculated MW |
| --- | --- | --- |
| ZScan20 | 3300.0 | 3299.7 |
| ZF422 | 3295.6 | 3294.7 |
| CP1 | 2905.8 | 2906.3 |
| ZF133-11 | 3113.1 | 3113.5 |
| ZF133-11 C7E | 3139.2 | 3139.4 |
| human MT2 | 6042.0 | 6042.3 |

**Table S3**. Determination of the *K*_d1_ value of Zn_7_MT2 by the competition with PAR in 50 mM HEPES buffer (*I* = 0.1 M from NaCl), pH 7.4, 25°C. 1.7 µM Zn_7_T2 was incubated with 2-400 µM PAR and the absorbance was monitored spectrophotometrically at 492 nm after 40 min. The reported values are the averages of three independent samples. The concentrations of [Zn(PAR)_2_] species and *K*_d1_ value were calculated using ε_492_ = 71,500 M^-1^⋅cm^-1^ and *K*_d12_^PAR^ = 7.08⋅10^-13^ M^2^, respectively.^1^

| PAR_total_  (μM) | A_492_ | [Zn(PAR)_2_] (μM) | [PAR]  (μM) | [Zn_6_T2]  (μM) | [Zn_7_T2]  (μM) | *K*_d1_  (M) | -log*K*_d1_ |
| --- | --- | --- | --- | --- | --- | --- | --- |
| 2 | 0.1200 | 0.168 | 1.66 | 0.168 | 1.53 | 4.70⋅10^-9^ | **8.33** |
| 5 | 0.0217 | 0.304 | 4.39 | 0.304 | 1.40 | 2.43⋅10^-9^ | **8.62** |
| 10 | 0.0325 | 0.454 | 9.09 | 0.454 | 1.25 | 1.42⋅10^-9^ | **8.85** |
| 25 | 0.0498 | 0.696 | 23.6 | 0.696 | 1.00 | 6.13⋅10^-10^ | **9.21** |
| 50 | 0.0665 | 0.930 | 48.1 | 0.930 | 0.770 | 3.43⋅10^-10^ | **9.46** |
| 100 | 0.0903 | 1.26 | 97.5 | 1.26 | 0.437 | 2.72⋅10^-10^ | 9.56 |
| 150 | 0.1032 | 1.44 | 147 | 1.44 | 0.257 | 2.66⋅10^-10^ | 9.58 |
| 200 | 0.1221 | 1.71 | 197 | 1.71 | *n.d.* | *n.d.* | *n.d.* |
| 300 | 0.1424 | 1.99 | 296 | 1.99 | *n.d.* | *n.d.* | *n.d.* |
| 400 | 0.1549 | 2.17 | 396 | 2.17 | *n.d.* | *n.d.* | *n.d.* |
| **Average *K*_d1_ value based on the first three points** | | | | | | 1.9 ± 1.8 ⋅10^-9^ | **8.9 ± 0.5** |

**Table S4**. Determination of the *K*_d1_ value of Zn_7_T2 by the competition with PAR in 50 mM HEPES buffer (*I* = 0.1 M from NaCl), pH 7.4, 25°C. 1.7 µM Zn_7_MT2 was incubated with 2-400 µM PAR and the absorbance was monitored spectrophotometrically at 492 nm after 40 min. The reported values are the averages of three independent samples. The concentrations of [Zn(PAR)_2_] species and *K*_d1_ value were calculated using ε_492_ = 71,500 M^-1^⋅cm^-1^ and *K*_d12_^PAR^ = 7.08⋅10^-13^ M^2^, respectively .^1^

| PAR_total_  (μM) | A_492_ | [Zn(PAR)_2_] (μM) | [PAR]  (μM) | [T]  (nM) | [Zn_7_T2]  (μM) | *K*_d17_^av^  (M^7^) | *K*_d_^av^  (M) | -log*K*_d_^av^ |
| --- | --- | --- | --- | --- | --- | --- | --- | --- |
| 2 | 0.1200 | 0.168 | 1.66 | 24.0 | 1.68 | 3.8 ⋅ 10^-54^ | 2.34⋅10^-8^ | **7.63** |
| 5 | 0.0217 | 0.304 | 4.39 | 43.4 | 1.66 | 5.6 ⋅ 10^-58^ | 6.63⋅10^-9^ | **8.18** |
| 10 | 0.0325 | 0.454 | 9.09 | 64.9 | 1.64 | 5.4 ⋅ 10^-61^ | 2.45⋅10^-9^ | **8.61** |
| 25 | 0.0498 | 0.696 | 23.6 | 99.4 | 1.60 | 2.6 ⋅ 10^-65^ | 5.95⋅10^-10^ | 9.23 |
| 50 | 0.0665 | 0.930 | 48.1 | 133 | 1.57 | 1.3 ⋅ 10^-68^ | 2.00⋅10^-10^ | 9.70 |
| 100 | 0.0903 | 1.26 | 97.5 | 180 | 1.52 | 7.8 ⋅ 10^-72^ | 6.94⋅10^-11^ | 10.16 |
| 150 | 0.1032 | 1.44 | 147 | 206 | 1.49 | 7.2 ⋅ 10^-74^ | 3.56⋅10^-11^ | 10.45 |
| 200 | 0.1221 | 1.71 | 197 | 244 | 1.46 | 4.9 ⋅ 10^-75^ | 2.42⋅10^-11^ | 10.62 |
| 300 | 0.1424 | 1.99 | 296 | 284 | 1.42 | 5.6 ⋅ 10^-77^ | 1.28⋅10^-11^ | 10.89 |
| 400 | 0.1549 | 2.17 | 396 | 309 | 1.39 | 1.9 ⋅ 10^-78^ | 7.90⋅10^-12^ | 11.10 |
| **Average *K*_d_^av^ value of the first three points** | | | | | | | 2.8 ± 1.7 ⋅10^-9^ | **8.1 ± 0.5** |

**Figure S1**. CD titration of 5 μM metal free ZF133-11 and ZF133-11_C/H_ zinc fingers with ZnSO_4_ in 20 mM Tris buffer (*I* = 0.1 M), pH 7.4, 25°C. Insets show ellipticity decrease monitored at 222 nm, which demonstrate Zn(II)-to-ZF 1:1 stoichiometry in both cases.

**Figure S2.** CD spectra of MT2 at various concentration (0.5-10 μM) used for equilibration with 5 μM metal free ZFs. Ellipticity of MT2 at 222 nm (red dash line) was subtracted from total ellpipticity of MT2 – zinc finger samples to obtain ellipticity of ZF only.

**Figure S3.** Examples of mass spectra obtained for samples with 30 µM apoMT2a and 30 µM apo zinc binding peptide in presence of various number of Zn(II) equivalents. CP1-2015 peptide (top row) is almost fully saturated as it has a very high affinity for Zn(II). ZF422 (middle row) actually competes with MT2 for binding of the Zn(II) as we see both the apo and metal saturated form. ZScan 20 peptide (bottom row) has relatively low affinity toward Zn(II), therefore it exists mostly in apo form.

**References**

1. A. Kocyła, A. Pomorski and A. Krężel, Molar absorption coefficients and stability constants of Zincon metal complexes for determination of metal ions and bioinorganic applications. *J. Inorg. Biochem.* 2017, 176, 53−65.
